# Overexpression of Nrf2 in bone marrow mesenchymal stem cells promotes B-cell acute lymphoblastic leukemia cells invasion and extramedullary organ infiltration through stimulation of the SDF-1/CXCR4 axis

**DOI:** 10.3389/fphar.2024.1393482

**Published:** 2024-07-16

**Authors:** Lin Zheng, Chengyun Pan, Dan Ma, Qin Shang, Tianzhen Hu, Tianzhuo Zhang, Qian Kang, Xiuying Hu, Shuyun Cao, Li Wang, Hong Luo, Jishi Wang

**Affiliations:** ^1^ Department of Hematology, Affiliated Hospital of Guizhou Medical University, Guiyang, China; ^2^ Department of Clinical Medical School, Guizhou Medical University, Guiyang, China; ^3^ Department of Guizhou Province Hematopoietic Stem Cell Transplantation Center and Key Laboratory of Hematological Disease Diagnostic and Treatment Centre, Guiyang, China; ^4^ Guizhou Provincial Engineering Technology Research Center for Chemical Drug R&D, Guizhou Medical University, Guiyang, China

**Keywords:** B-acute lymphocytic leukemia, MSCs, Nrf2, SDF-1/CXCR4, extramedullary infiltration

## Abstract

**Background:**

Tumor microenvironment (TME) represents the key factor inducing leukemia development. As stromal cells within the leukemia microenvironment, Bone Marrow Mesenchymal Stem Cells (BM-MSCs) can trigger leukemia progression under certain conditions. As a critical transcription factor, nuclear factor erythroid related factor 2 (Nrf2) can modulate antioxidant response and antioxidant enzyme gene expression, and prevent various oxidative changes. We previously identified a novel mechanism by which Nrf2 promotes leukemia resistance, providing a potential therapeutic target for the treatment of drug-resistant/refractory leukemias. However, the role of Nrf2 in BM-MSCs from B-cell acute lymphoblastic leukemia (B-ALL) patients has not been clearly reported. The present work focused on investigating the effect of Nrf2 overexpression within MSCs on leukemia cell invasion, extramedullary infiltration and proliferation as well as its downstream pathway.

**Methods:**

Through clinical sample detection, *in vitro* cell experiments and *in vivo* animal experiments, the role of Nrf2 within MSCs within adult B-ALL cell migration and invasion and its potential molecular mechanism was explored through transcriptome sequencing analysis, RT-PCR, Western blot, cell migration, cell invasion, lentivirus transfection and other experiments.

**Results:**

Nrf2 was highly expressed in BM-MSCs from patients with B-ALL as well as in BM-MSCs co-cultured with leukemia cells. Overexpression of Nrf2 within MSCs significantly promoted leukemia cell migration, invasion and proliferation. The extramedullary organ infiltration rate in B-ALL model mice receiving the combined infusion of both cell types dramatically increased relative to that of leukemia cells alone, accompanied by the significantly shortened survival time. Mechanism study found that Nrf2 overexpression within MSCs promoted PI3K-AKT/ERK1/2 phosphorylation in the downstream pathway by activating SDF-1/CXCR4 axis, ultimately leading to extramedullary infiltration of leukemia cells.

**Conclusion:**

High Nrf2 expression with in MSCs enhances leukemia cell invasion and migration, which then accelerates infiltration in leukemic extramedullary organs. Targeting Nrf2 or inhibiting its downstream signal molecules may be the effective interventions for B-ALL patients treatment.

## 1 Introduction

ALL, a highly invasive hematological cancer, occupies about 80% of childhood leukemia and 20% of adult leukemia (Short and Kantarjian, 2023). Over the last few decades, medical progresses greatly improve the survival rate in B-cell acute lymphoblastic leukemia (B-ALL) adult patients (Cruz et al., 2021; Iacobucci et al., 2023). Advances in treatment have elevated the survival rate of children to over 80%, but just 30%–40% of adults can attain long-time disease-free survival (Haas and Borkhardt, 2022). Residual leukemic stem cells and progenitor cells have posed a persistent risk of disease recurrence and are a major challenge for all treatments (Creasey et al., 2021). Therefore, it is an important goal to develop novel drugs and methods to treat recurrence for the sake of improving the cure rate (Hong et al., 2021; Barz et al., 2022; Tran and Hunger, 2022).

Previous studies on tumors mainly focus on tumor cell proliferation, infiltration and migration (Plakhova et al., 2023). Recently, it has been reported that tumor microenvironment (TME) has changed from tumor bystander and promoter to an essential fateful role (Xu et al., 2021). TME includes all the structures that recruit the organ, including blood vessels, immune infiltration, fibroblasts and extracellular matrix (Xia et al., 2020). Tumor progression, treatment resistance, invasion and metastasis are the characteristics of two-way interaction between cancer cells and TME (Sahinbegovic et al., 2020). Additionally, the crosstalk between cancer cells and their environment involves mutual paracrine and paracrine signal transduction via many different pathways (Kumar et al., 2018). The bone marrow hematopoietic microenvironment is in a dynamic evolutionary process and has a very important role in the survival and growth of leukemia cells (Corn et al., 2020), mainly in the promotion of invasion and infiltration of hematological tumors (Guo et al., 2022). Bone marrow hematopoietic microenvironment has an essential effect on protecting against leukemia genesis, development, migration and relapse, suggesting that hematopoietic microenvironment can be the possible intervention target (Lou et al., 2022). Bone marrow hematopoietic microenvironment, the dynamic and complex biological tissue, is comprised by cellular and non-cellular components (Fallati et al., 2022). Its cellular components include HSC and Sertoli cells, like adipocytes, osteoblasts, mesenchymal stem cells (MSCs), unmyelinated Schwann cells, sympathetic neurons, nerve cells, and endothelial cells (Tan et al., 2021). In particular, MSCs enhance leukemic cell survival and protect them from chemotherapy (He et al., 2021).

Drug resistance among patients with B-ALL, especially in patients with relapse refractory B-ALL, has been widely suggested to be closely related to the bone marrow microenvironment (Fathi and Vietor, 2021). Traditional chemotherapy usually achieves the treatment purpose by removing a large number of cloned cells (Dander et al., 2021). However, B-ALL cells hidden in bone marrow niches can escape the killing effect of chemotherapeutic drugs, which is tightly associated with the signals of survival and drug resistance received by these cells from bone marrow stromal cells (Balandrán et al., 2021). Therefore, there is an urgent need to analyze how MSCs interact with B-ALL cells and how they contribute to B-ALL progression (Witkowski et al., 2020).

CXCR4, the 7-transmembrane G-protein coupled receptor (GPCR), shows high expression within numerous cells including endothelial cells, lymphocytes, stromal fibroblasts, hematopoietic stem cells, and tumor cells (Yang et al., 2023). Stromal cell-derived factor-1 (SDF-1 or CXCL-12) is a member of cell surface receptor family and is a ligand of CXCR-4, moreover, it is mainly produced via adult bone marrow stromal cells. SDF-1/CXCR-4 signal transduction has a critical effect on numerous physiopathological processes. For instance, SDF-1/CXCR4 modulates epithelial-mesenchymal transformation (EMT) of sacral chondrosarcoma, glioblastoma, and oral squamous cell carcinoma SDF-1/CXCR-4 signal transduction also regulates actin polymerization, vascular endothelial cell adhesion and migration to BM-MSCs (Gao et al., 2021; Jia et al., 2023). However, it remains unclear whether SDF-1/CXCR4 axis mediates the interaction between Nrf2 overexpression in MSCs of B-ALL and leukemia cells.

In this study, we specifically elucidated whether the expression of Nrf2 in MSCs is crucial for leukemia cell migration and invasion, examine if SDF-1/CXCR4 signal axis is activated by Nrf2 overexpression in MSCs, and analyze whether it is the key signal axis between Nrf2 overexpressed MSCs and ALL for promoting leukemia cell invasion, which provides a basis for further exploring the potential downstream molecular mechanisms of interaction between Nrf2-overexpressing MSCs and B-ALL cells.

## 2 Materials and methods

### 2.1 Clinical samples and cells

Using a simple random sampling method, eighty myeloid blood samples were obtained in relapsed B-ALL patients from Affiliated Hospital of Guizhou Medical University from September 2021 to May 2023. Meanwhile, normal samples were donated by healthy individuals. Supplementary Table S1 displays the patient clinical data. Our study protocols gained approval from Research Ethics Committee. Sample collection was completed under informed consent. Ficoll-Hypaque density centrifugation was conducted to purify the original patient samples, and samples were later analyzed using RPMI-1640 medium that contained 10% fetal bovine serum (FBS) together with 1% penicillin-streptomycin under 37°C with 5% CO2.

Human cells (including Nalm-6/RS4; 11 cells) were provided by Leibniz Institute DSMZ - German Conservation Center for Microbiology and Cell Cultures (DSMZ), assessed for *mycoplasma* pollution and verified by short tandem repeat characterization, and cultivated within RPMI-1640 that contained 10% FBS as well as 1% penicillin-streptomycin under 37°C with 5% CO2.

### 2.2 MSCs separation and culture

To collect bone marrow blood for separating BM-MSCs, we obtained bone marrow blood from 35 patients with relapsed B-ALL for our experiments after obtaining informed consent. Then, BM-MSCs were isolated from each sample by gradient centrifugation of lymphocyte separator, and cells at the 2-4 cytosolic stages were chosen for further experiments. Afterwards, MSCs were cultured in the mature BM-MSC whole medium (Shanghai Saipai Biotechnology Co, Ltd.).

### 2.3 Co-culture system

To establish a two-dimensional (2D) system for co-culture, we introduced ALL cells at a 4:1 ratio and then co-cultured 1 × 10^5^/mL BM-MSCs on culture plates. We co-cultured MSCs with leukemia cells for a 72-h period according to previous description (Bonilla et al., 2019). For every separate replicate experiment, we cultured MSCs obtained in one patient separately as experimental controls. To collect leukemia cells suspended within the co-culture system, we carefully removed Nalm-6/RS4; 11 cells from MSCs monolayer. Leukemia cells adhering onto MSCs surface were removed by extensively washing the co-culture system with PBS-EDTA1.

### 2.4 Cell migration and invasion

To analyze cell migration and invasion (Transwell chambers with and without Matrigel, respectively), the bottom Transwell chamber supplemented with MSCs (1 × 10^5^/mL) was incubated overnight, and the top Transwell chamber was added Nalm-6/RS4; 11 cells (4 × 10^5^/mL) (pore diameter, 8.0 μm; Corning Incorporated, Costar). Following 24-h incubation, those migrating and invasive cells within the bottom chamber were counted with the inverted microscope. Besides, photos of ×200 magnification were obtained, with five fields of views being chosen at random migrating and invasive cells. Quantification of migrating and invading cells is achieved by direct counting using a cell counter.

### 2.5 Lentivirus transfection

Human Nrf2 silenced RNA (SI-Nrf2) and Nrf2 overexpressed lentivirus particles (LV-Nrf2) were offered by Genechem Co, Ltd. (Shanghai, China). In this study, Si-Nrf2/LV-Nrf2 was transfected in MSCs with reference to specific protocols. Empty vector (EV)-transfected MSCs served as control cells. The stable MSCs were obtained following amplification and maintenance in RPMI-1640 containing 10% FBS for a 5-day period. Thereafter, MSCs expressing SI-Nrf2/LV-Nrf2 were screened by puromycin (2 μg/mL).

### 2.6 Flow cytometry analysis

Qualitative and quantitative levels of various markers were detected by multiparametric flow cytometry. To be specific, samples were stained with different combinations of antibodies against CD11b, CD45, CD105, CD22, CD90, and CD34 (Supplementary Table S2). Afterwards, cells were stained with appropriate surface antibodies, followed by fixation and 20-min permeabilization using Cytofix/Cytoperm reagent (BD Biosciences), and staining using intracellular molecular antibodies.

Subsequently, flow cytometry was conducted to monitor ALL cells *in vivo*. Myeloid blood or peripheral blood was collected to prepare single cell suspension, which was then treated using blocking buffer (5% human serum; Sigma-Aldrich), anti-human CD22-PE antibody (Stemcell Technologies) together with anti-human CD45PE-Cy-7 antibody (eBioscience, San Diego, CA, USA). BD FACS lysate was utilized for the lysis of red blood cells. At last, cells were rinsed with 0.9% saline and resuscitated. All acquisitions were carried out using the FACSLyric device (BD Biosciences), while BD FACSuite v1.3 software (BD Biosciences) and Flowjo software (TreeStar) were employed for data analysis.

### 2.7 Real-time quantitative polymerase chain reaction (RT-PCR)

We utilized the RNeasy kit (Qiagen, Hilden, Germany) for total RNA extraction, which was then prepared into cDNA through reverse transcription using Omniscript reverse transcription kit (Qiagen). Thereafter, by using iQ SYBR Green Supermix (Bio-Rad, Hercules, CA, USA) and primers, RT-PCR was conducted in line with specific protocols to analyze cDNA. By adopting β-actin as the endogenous reference, target gene expression was measured through comparative CT (2-dimensional CT) method. Human primers (Generay Biotech Co, Shanghai, China) below were utilized (Supplementary Table S3).

### 2.8 Western blotting

Both stromal and leukemia cells were gathered and cleaved with RIPA cleavage buffer containing 1% phenylmethyl sulfonyl fluoride (PMSF) (Beyotime, Shanghai, China). Thereafter, this work applied SDS-PAGE for separating 10–30 μg protein and transferred proteins on PVDF membrane. Then, 5% skin milk was added to block the membrane for 1–2 h, followed by overnight primary antibody incubation under 4°C. Protein antibodies (1:1000, AKT, p-AKT, ERK, p-ERK, CXCR4, Nrf2, and β-Actin antibodies) and β-actin antibody (1:3000) were used. Afterwards, we added Tris-Buffered Saline and Tween 20 (TBST) to rinse the membrane, followed by another 1-h secondary antibody incubation (1 TBST). Protein expression was subsequently detected with the ECL reagent. Finally, the band gray values were examined by ImageJ software, with β-actin being an internal reference.

### 2.9 Apoptosis

After collection, cells were washed by PBS prior to staining using membrane-bound protein-V/propidium iodide (PI) following specific instructions (7Sea Pharmatech, Shanghai, China) for determining apoptosis rate.

### 2.10 β-galactosidase staining

For BM-MSC or BM-MSC co-cultured with RS4; 11/Nalm-6, β-galactosidase staining was performed in 6-well plates after washing with PBS according to specific guidelines. The blue-stained cell percentage (senescent cells) was observed using the microscope and photographed (200 ×). The senescent cell percentage of every group was assessed from five randomized fields. In addition, the average level was calculated to analyze cell senescence in every group.

### 2.11 Xenograft tumor model

The Animal Protection and Welfare Committee of Guizhou Medical University (No.1900779) approved our mouse xenograft experiment. During animal study, 4-6-week-old female mice developing non-obese diabetes/severe combined immunodeficiency (NOD/SCID) were randomized as four groups (n = 5 in each group). Mice were then given intravenous injection of RS4; 11 cells alone, or RS4; 11 cells combined with MSCs, or RS4; 11 cells plus MSCs-EV, or RS4; 11 cells plus MSCs-LV-Nrf2 (1 × 10^6^ MSCs and 4 × 10^6^ RS4; 11 cells) through tail vein. Peripheral blood was obtained from mouse at random, meanwhile, human ALL cells were detected at 4 and 6 weeks. To carry out histopathological analysis, the bone marrow, liver, spleen and femur sections were subjected to fixation with 10% tissue fixation solution, paraffin embedding, and hematoxylin and eosin (H&E) staining. We then gathered bone marrow cells from both femurs. Various antibodies were added to label single cell suspension, and flow cytometry was conducted to analyze human cell percentage.

### 2.12 Immunohistochemical (IHC) staining

Primary and secondary antibodies were diluted at ratios identical to those used in IF analysis. Thereafter, the positive cell staining degree was multiplied by positive cell proportion to determine the staining index for IHC staining. To be specific, the score of positive cell staining degree was determined by multiplying stained cell number by cell color depth score (0–3 indicating no staining, light yellow, tan, and brown, separately; dint 0, Magi1, Magi2, and prime three representing positive cell proportions of 0%–5%, 5%–25%, 25%–50%, 50%–75% and >75%, respectively). IHC staining was carried out following relevant operating instructions. Cells (tissues) were osmotically fixed with Triton X-100, with 20% sodium citrate being adopted in antigen retrieval. Thereafter, cells (tissues) were sealed with goat serum for a 60-min period, and incubated using antibody under 4°C overnight. The experimental results were determined based on positive cell percentage within each tissue and positive cell staining intensity.

### 2.13 H&E analysis for B-ALL cell invasion *in vivo*

Mouse spleen, liver, and femur samples in each group were taken for fixation with 4% paraformaldehyde, paraffin embedding and H&E staining to observe morphological changes with a light microscope.

### 2.14 Reagents and antibodies

AMD3100, provided by MedChemExpress (Shanghai, China), was prepared with anhydrous ethanol at a 100 mM storage concentration and diluted with RPMI-1640 to prepare the 10 mM working solution. Antibodies against AKT, phospho-AKT, ERK and phosphor-ERK were provided by Cell Signaling Technology (USA). CXCR4 antibodies were provided by Solibao Biotechnology Co., Ltd. (Beijing). In Western blotting, the secondary antibody utilized was provided by Medical Discovery Lida (Beijing, China). Alexa Fluor™ 488 donkey anti-rabbit IgG (H + L) fluorescent secondary antibody was obtained from Invitrogen of Thermo Fisher Scientific (USA). Goat anti-mouse IgG (H + L) and CoraLite594-coupled secondary antibodies were provided by Wuhan Proteintech Group of China. Electrochemiluminescence (ECL) reagents for Western blotting were provided by Shanghai Qihai Pharmaceutical Co., Ltd.

### 2.15 Transcriptome sequencing

Those treated MSCs-LV-Nrf2 and MSCs-EV were gathered for transcriptome sequencing analysis, respectively. After lysis of cells using Trizol reagent, we sent samples to Hangzhou Lianchuan Biotechnology Company to conduct transcriptome sequencing analysis. Sequencing process is detailed in Supplementary Methods S1.

### 2.16 Statistical analysis

Data were analyzed with SPSS 20.0. Experimental data were repeated thrice. Independent Student’s t-test (paired or unpaired double tails) was adopted in univariable regression. Multiple groups were analyzed with one-way analysis of variance (ANOVA), while results were presented by mean ± SEM. Survival data were explored by Log-ranch test. Statistical analysis was conducted with PRISM V8.0 (GraphPad Software, San Diego, California). *p* < 0.05 stood for statistical significance.

## 3 Results

### 3.1 High expression of Nrf2 in relapsed B-ALL-MSCs and altered biology of MSCs under co-culture conditions

**FIGURE 1:**
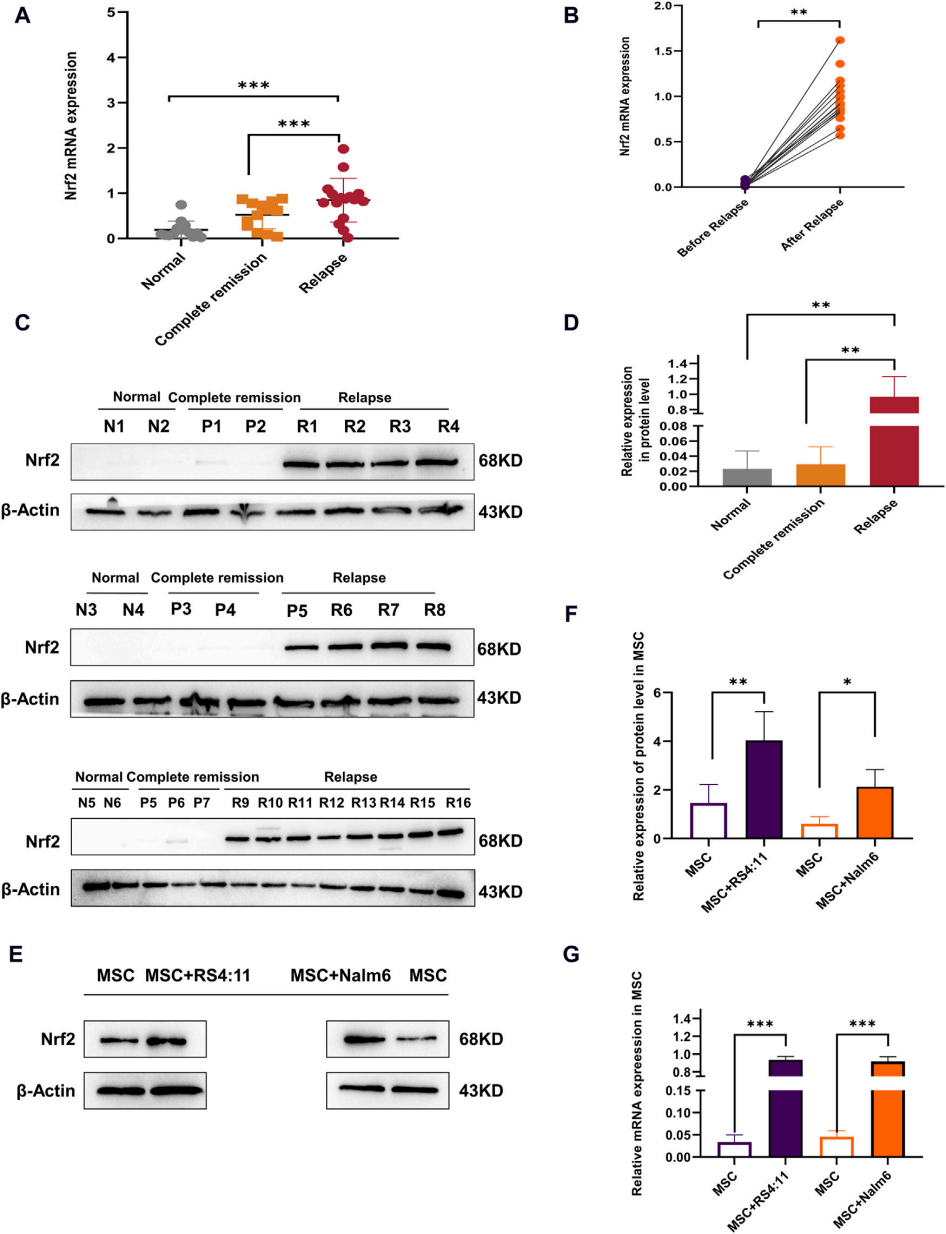
Overexpression of Nrf2 in relapsed B-ALL patients and altered biology of MSCs in the leukemia microenvironment **(A)** Nrf2 levels were measured by RT-PCR within normal (n = 20), complete remission (n = 20), and relapsed (n = 35) B-ALL patients. **(B)** Nrf2 levels before and following relapse among same B-ALL patients by RT-PCR method. **(C)** Nrf2 levels among normal donors (n = 6), complete remission (n = 7) and relapsed (n = 16) B-ALL patients was measured through Western blotting. **(D)** Histogram of Western blotting statistics. **(E)** Nrf2 levels within MSCs single culture system and MSCs + Nalm-6/RS4; 11 co-culture system were measured through Western blotting. **(F)** Relative gray value of Nrf2 level. **(G)** Nrf2 mRNA levels within MSCs single culture system and MSCs + Nalm-6/RS4; 11 co-culture system were measured through RT-PCR. Every assay was conducted thrice independently and represented by average soil SD. *p < 0.05, **p < 0.01, ***p < 0.001.

Nrf2 expression was identified within bone marrow mesenchymal stem cells from healthy donors, complete remission and relapsed B-ALL patients (Figure 1A). Nrf2 mRNA level in MSCs of the relapse group (n = 35) was significantly higher than normal control (n = 20) and complete remission groups (n = 20). Among these B-ALL patients, Nrf2 mRNA level in MSCs significantly increased following relapse compared with that before (Figure 1B). As revealed by Western blotting, Nrf2 in MSCs levels among relapsed B-ALL patients increased relative to healthy donors and patients with complete remission (Figures 1C, D). This study further established a simulated bone marrow microenvironment *in vitro* and Immunophenotype identification MSCs as well as differentiation capacity for lipogenic osteogenesis (Supplementary Figures S1A, B). Co-cultured MSC showed a significant increase in senescence compared to mono-cultured MSC (Supplementary Figure S1C). Interesting co-cultured MSCs with Nalm-6/RS4; 11 cells to detect Nrf2 expression (72-h co-culture bone marrow MSCs with leukemia cells). Based on Western blotting results, Nrf2 expression in MSCs co-cultured with Nalm-6/RS4; 11 cells dramatically tended to increase relative to that in MSCs culture alone (Figures 1E, F). Consistently, RT-PCR assay revealed the evidently increased Nrf2 expression in MSCs + Nalm-6/RS4; 11 co-culture system relative to the single culture of MSCs (Figure 1G). Consequently, Nrf2 upregulation is closely related to B-ALL relapse, in addition, the biological properties of MSCs were altered to varying degrees under co-culture conditions with leukemia.

### 3.2 Nrf2 in BMSCs promotes B-ALL cell migration and invasion

**FIGURE 2:**
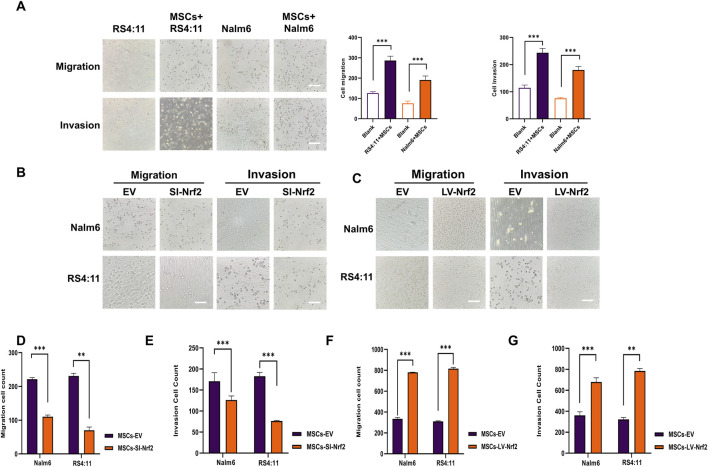
Nrf2 in BMSCs promotes B-ALL cell migration and invasion **(A)** Transwell assays performed for analyzing cell invasion and migration after incubation with Nalm-6/RS4; 11 alone and co-culture with MSCs for 24 h **(B, D, E)** Downregulation of Nrf2 expression in MSCs was tested for migration and invasion rates of Nalm6/RS4:11 cells in the microcellular compartment at 24 h. Migration and invasion rates were significantly decreased in the downregulated group (p < 0.01). **(C,F,G)** Upregulation of Nrf2 expression in MSCs revealed a significant increase in the migration and invasion rate of Nalm6/RS4: 11 in cells within the upregulated group at 24 h. Each assay was conducted thrice independently and denoted as mean ± SD. *p < 0.05, **p < 0.01, ***p < 0.001.

Based on the above results, we verified the differences in Nrf2 expression in cultured MSCs alone and co-cultured MSCs. Therefore, we need to further explore the potential impact of Nrf2 expression in bone marrow MSCs on the biological functions of B-ALL cells. Among them, migration and invasion of leukemia cells are important bases for malignant proliferation of tumor cells (Pan et al., 2021). To be specific, leukemia cells of pre-laid MSCs group showed markedly increased migration and invasion abilities relative to blank group (Figure 2A). We successfully transfected Nrf2 silencing (MSCs-SI-Nrf2)/overexpressing lentiviral (MSCs-LV-Nrf2) particles in MSCs and verified by Western blotting after 72-h co-culture with Nalm-6/RS4; 11 (Supplementary Figure S1D). When Nrf2 expression decreased in MSCs, Nalm-6/RS4; 11 cell invasion and migration abilities decreased (Figures 2B, D, E). Interestingly, when Nrf2 was overexpressed in MSCs, migration and invasion of leukemia cells were enhanced (Figures 2C, F, G). We found that altered levels of Nrf2 in MSCs significantly affect the migration and invasive ability of leukemia cells.

### 3.3 CXCR4 overexpression in B-ALL cells was positively related to Nrf2

**FIGURE 3:**
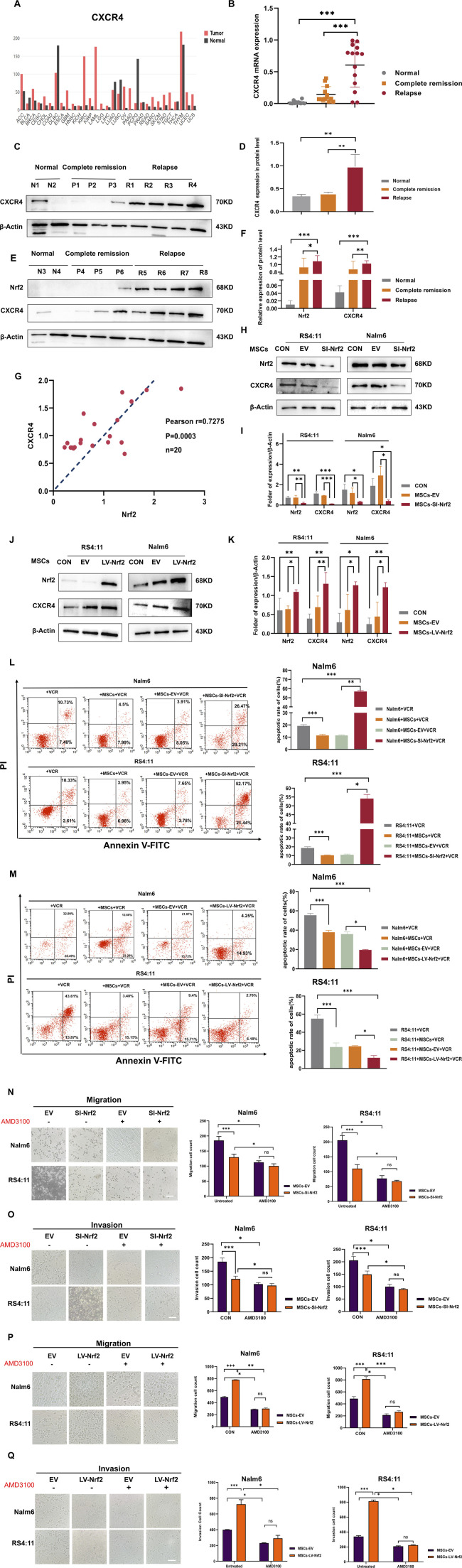
CXCR4 overexpression in B-ALL cells was positively related to Nrf2 **(A)** GEPIA database analysis for CXCR4 levels within tumor and non-carcinoma tissues. **(B)** RT-PCR assay on CXCR4 levels within healthy (n = 10), complete remission (n = 20) and relapsed (n = 20) B-ALL patients. **(C,D,E,F)** Western blotting was conducted for detecting CXCR4 and Nrf2 protein levels among healthy (n = 4), complete remission (n = 6) and relapsed B-ALL patient (n = 8) samples. **(G)** Association of Nrf2 with CXCR4 in B-ALL was measured through RT-PCR (n = 20, r = 0.7275 = 0.5232, p = 0.0003). **(H,I)** Western blotting was performed to detect the expression of CXCR4 in RS4; 11/Nalm-6 in the co-culture system after downregulation of Nrf2 in MSCs. **(J,K)** Western blotting was performed to detect the expression of CXCR4 in RS4; 11/Nalm-6 in the co-culture system after upregulation of Nrf2 in MSCs. **(L)** MSCs-SI-Nrf2 significantly affected leukemia cell sensitivity to vincristine. **(M)** MSCs-LV-Nrf2 had a similarly significant change in changing leukemia cell sensitivity to vincristine. **(N–Q)** Transwell tests were performed to detect the number of Nalm-6 and RS4; 11 cells migrating and invading in the MSCs-EV, MSCs-SI-Nrf2, MSCs-LV-Nrf2, and treated AMD3100 (20 μM, upper chamber) groups, respectively, after 24 h of cell incubation. Each assay was conducted thrice independently and denoted as mean ± SD. *p < 0.05, **p < 0.01, ***p < 0.001.

Since SDF-1/CXCR four axis is thought to be an important signaling axis mediating communication between tumor cells and MSCs (Zhang et al., 2022). We analyzed whether regulating Nrf2 expression in MSCs enhanced ALL cell invasion and migration by SDF-1/CXCR4 axis. To investigate if CXCR4 upregulation had certain biological effects, this work assessed the levels of CXCR4 within tumor and non-carcinoma tissues based on GEPIA database (http://bioinformatics.psb.ugent.be/webtools/Venn/). We found that CXCR4 levels significantly increased in several tumors (Figure 3A). First, CXCR4 mRNA levels notably increased among patients with relapsed B-ALL compared with normal donors and those with complete remission (Figure 3B). Additionally, based on Western blotting assay, CXCR4 levels markedly increased among relapsed B-ALL patients compared with normal healthy donors versus those in complete remission (Figures 3C–F). RT-PCR assay demonstrated that Nrf2 expression was positively related to CXCR4 expression in B-ALL (r = 0.7275) (Figure 3G). These results suggest that CXCR4 expression is positively correlated with ALL recurrence and Nrf2 expression. Meanwhile, we further explored that when Nrf2 expression was downregulated in MSCs, CXCR4 expression within Nalm-6/RS4; 11 was reduced after 72-h co-culture of MSCs and Nalm-6/RS4; 11 (Figures 3H, I). In contrast, CXCR4 expression in Nalm-6/RS4; 11 was highly upregulated after MSCs-LV-Nrf2 transfection (Figures 3J, K). And we noted a significant elevation of SDF-1 levels in MSCs in the LV-Nrf2 group (Supplementary Figure S1E). We also found that changes in Nrf2 expression in MSCs had an effect on the sensitivity of leukemia cells to vincristine through functional assay results (Figures 3L, M).

In the next experiment after adding AMD3100 into the top Transwell chamber for blocking SDF-1/CXCR4 signaling axis, Nalm-6/RS4; 11 cell invasion and migration capacities decreased. Whereas there was no statistically significant difference between the EV group and the downregulated group (Figures 3N, O). When we further upregulated Nrf2 expression in MSCs, the assay revealed that the migration and invasion ability of leukemia cells was blocked after the same action with AMD3100, and there was no statistical difference between the EV group and the upregulated group (Figures 3P, Q). Preliminary evidence shows that when specifically blocking the SDF-1/CXCR4 signaling axis, the migration and invasion ability of leukemia cells can be effectively attenuated, and the changes of Nrf2 in MSCs did not affect the blocking results of the cells. Interestingly, AMD3100 incorporation into the co-culture system was found to similarly attenuate the proliferative effects of upregulated Nrf2 (Supplementary Figure S3). Collectively, SDF-1/CXCR four signaling axis is the vital factor related to impact of Nrf2 overexpression in MSCs on promoting leukemia cell invasion, migration and proliferation, and CXCR4 inhibitor AMD3100 can effectively block this process (Supplementary Figure S4).

### 3.4 Significant changes in gene expression profiles of leukemic cells in the leukemic microenvironment by Nrf2 overexpression in MSCs

**FIGURE 4:**
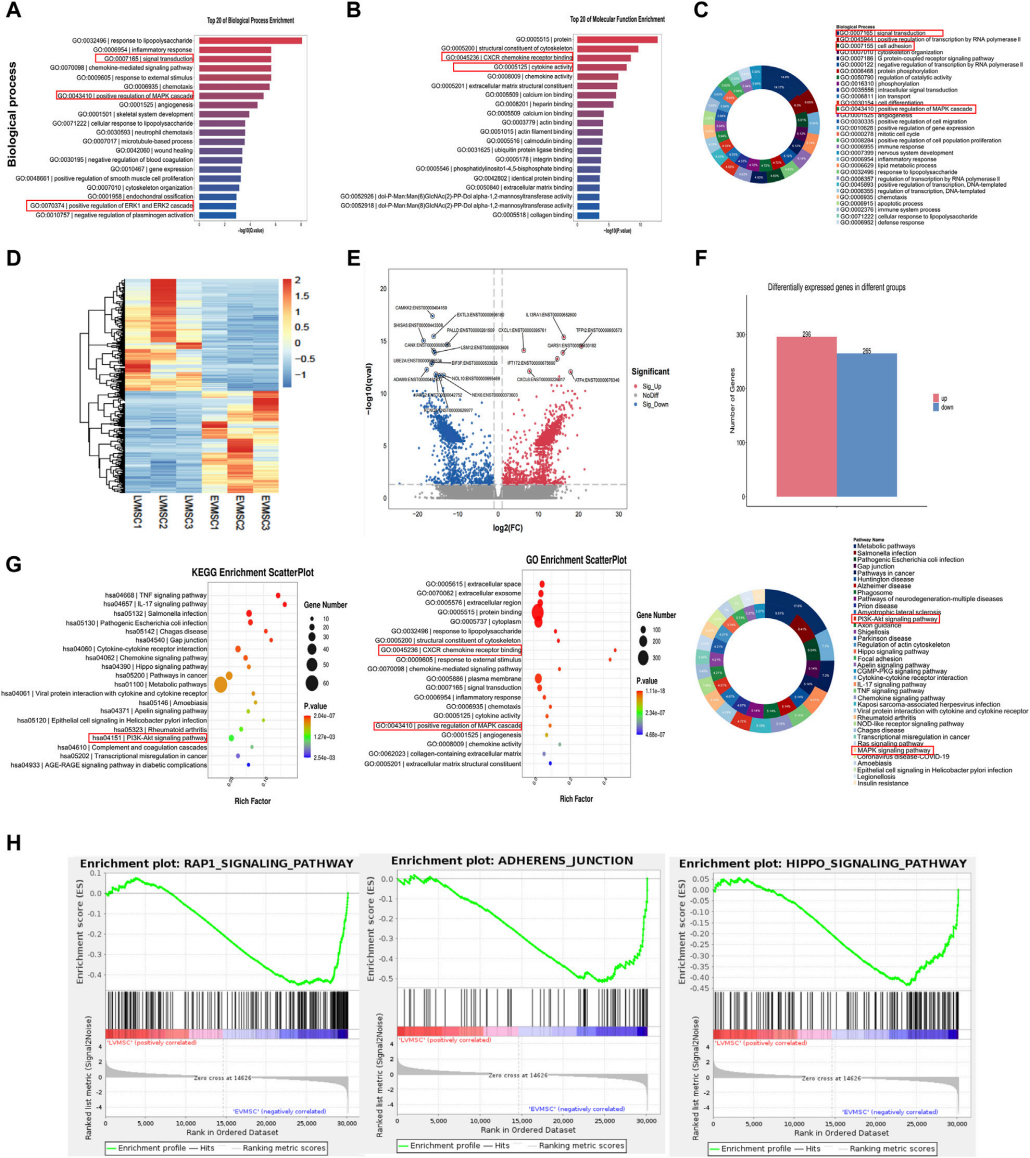
Significant changes in gene expression profiles of leukemic cells in the leukemic microenvironment by Nrf2 overexpression in MSCs **(A)** The top 20 biological processes enriched suggested the significant enrichment of positive regulation of MAPK cascade reaction, and positive regulation of ERK1/2 cascade reaction. **(B)** The top 20 molecular functions enriched. **(C)** Enrichment of biological processes. **(D)** The unsupervised clustering of DEGs observed from the volcano map. In the heatmap, FPKMs were normalized to row z scores. **(E)** In the volcano plot, blue and red stand for down- and upregulated DEGs relative to control MSCs (| Log2FC | ≥ 1, Q ≤ 0.05). **(F)** Compared with the control MSC, there were 561 DEGs in LV-Nrf2-MSC, including 296 with upregulation whereas 265 with downregulation. **(G)** Bubble diagrams involving DEG signals from MSCs transfected with lentivirus-empty vectors and LV-Nrf2. We selected PI3K-AKT and ERK1/2 pathways for further analysis. **(H)** Gene cluster enrichment analysis showed adhesion junctions associated with adhesion, migration and invasion of MSCs-LV-Nrf2, enrichment of RAP1 signal and hippopotamus signal.

In addition to characterizing the cellular function of MSCs-LV-Nrf2 and its effects on leukemia, RNA-sequencing was conducted to demonstrate the alterations of Nrf2 expression in controls and MSCs-LV-Nrf2 at the transcriptome level. Biological enrichment analysis of the top 20 signaling pathways included positive regulation of MAPK cascade response, and positive regulation of ERK1/2 cascade response (Figure 4A and Supplementary Figure S2). Typically, enrichment of those top 20 molecular functions and biological processes suggested that MSCs-LV-Nrf2 promoted genes related to leukemia cell adhesion, invasion and migration (Figures 4B, C). According to our main analysis, Nrf2 showed differential expression between control MSCs and MSCs-LV-Nrf2 in the transcriptional group. A volcano map was plotted to show the unsupervised clustering of differentially expressed genes (DEGs) (Figure 4D). There were altogether 561 DEGs discovered from differential expression analysis. Compared with the control MSCs, 296 genes showed upregulation, whereas 265 showed downregulation in MSCs-LV-Nrf2 group (Figures 4E, F). For investigating molecular mechanisms of Nrf2 overexpression within MSCs in leukemia cell invasion and migration, the transcriptional profiles of LV-Nrf2 lentivirus-transfected MSCs were detected and analyzed using RNA-Seq technology. As a result, the top 20 KEGG pathways were significantly enriched in PI3K-AKT, IL-17, AGE-RAGE, RAPI, ADHERENS-JUNCTION, HIPPO, and MAPK. In addition, pathway activation was extensively related to cell-to-cell signaling, adhesion junctions, and biological functions related to migration and invasion (Figures 4G, H).

### 3.5 PI3K/AKT and ERK pathways activation mediated by Nrf2 overexpression within MSCs significantly affected B-ALL cell migration and invasion

**FIGURE 5:**
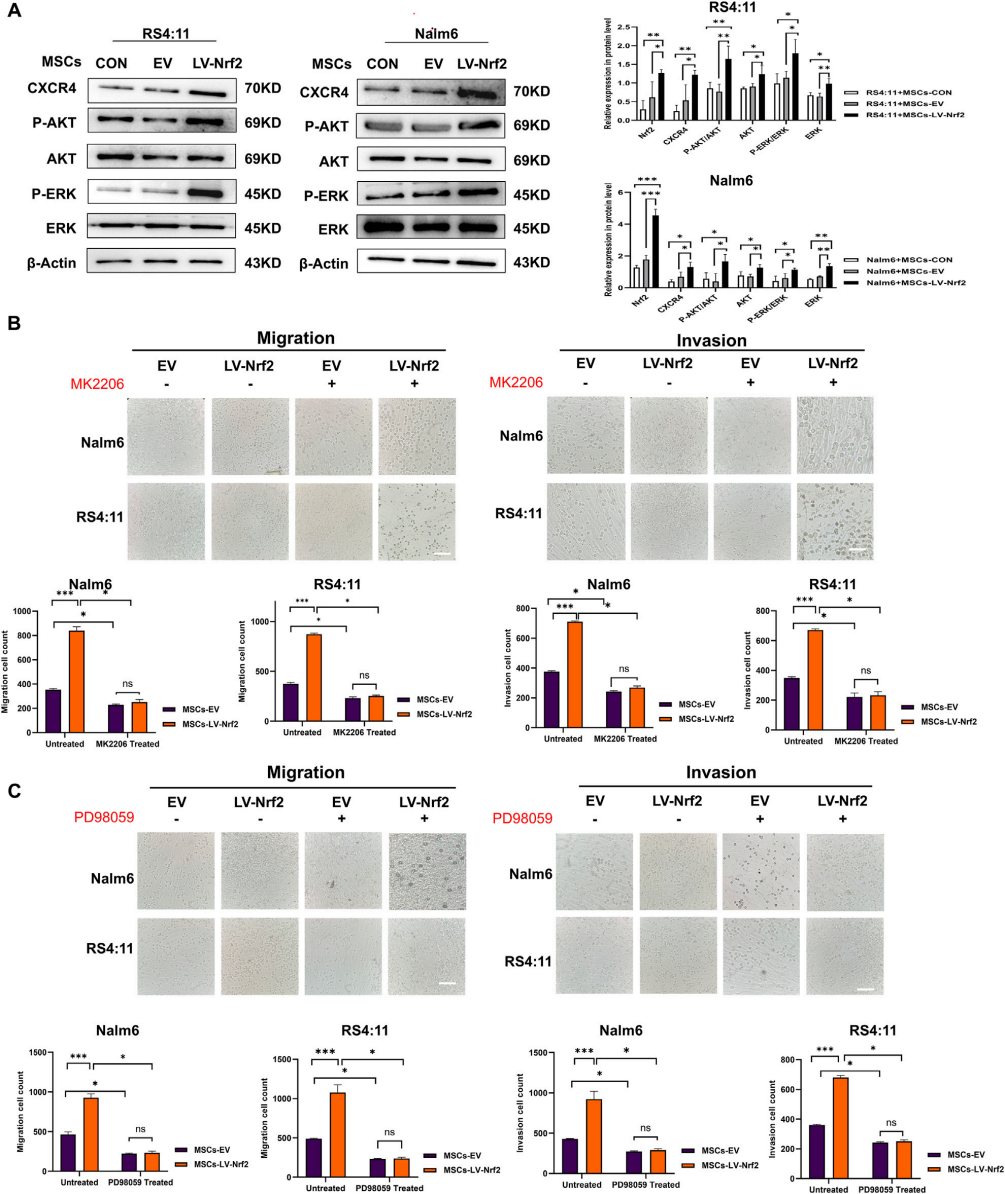
PI3K/AKT and ERK pathway activation mediated by Nrf2 overexpression in MSCs made a vital impact on B-ALL cell invasion and migration. **(A)** PI3K/AKT and ERK pathway in MSCs-LV-Nrf2 and RS4; 11/Nalm-6 co-culture conditions was evaluated through Western blotting. Histogram showed protein level quantification. **(B)** Addition of AKT inhibitor (MK2206), the results showed that the migration and invasion ability of leukemia cells were attenuated, and there was no statistically significant difference between the EV and LV-Nrf2 groups. **(C)** Addition of ERK inhibitor (PD98059) to the upper compartment also significantly blocked the migration and invasion ability of leukemia cells, with no statistically significant difference between the EV and LV-Nrf2 groups. Each assay was conducted thrice independently and represented by average soil SD. *p < 0.05, **p < 0.01, ***p < 0.001.

Previous studies have reported that specific binding of the SDF-1/CXCR4 axis activates phosphorylation of multiple downstream signaling pathways to promote tumor cell survival. Among them, ERK and PI3K/AKT signaling pathways are important in regulating migration and invasion of leukemia cells (Ladikou et al., 2020; Yang et al., 2023). Therefore, we initially chose the PI3K/AKT and ERK1/2 pathways as the study pathways. Subsequently, we performed Western blotting analysis (Figure 5A), and the assay results revealed that after upregulation of Nrf2 in MSCs and co-culture with leukemia cells, we found that the expression levels of PI3K/AKT and ERK pathways downstream of the SDF-1/CXCR4 axis, which are significantly associated with migration and invasion, tended to increase in leukemia cells. Then, we added AKT inhibitors and ERK inhibitors (MK2206 and PD98059) to the chambers to test whether the migration and invasion ability of leukemia cells would be blocked, and the results showed that (Figures 5B, C), the migration and invasion ability of leukemia cells in the chambers were attenuated after the addition of MK2206 and PD98059 treatment. This also shows that overexpression of Nrf2 in MSCs could promote the phosphorylation of the downstream pathways through activation of the SDF-1/CXCR4 axis, which led to a series of cascade reactions and promoted the migration and invasion of leukemia cells.

### 3.6 Overexpression of Nrf2 in MSCs promoted B-ALL cell infiltration in extramedullary organ through *in vivo* experiments

**FIGURE 6:**
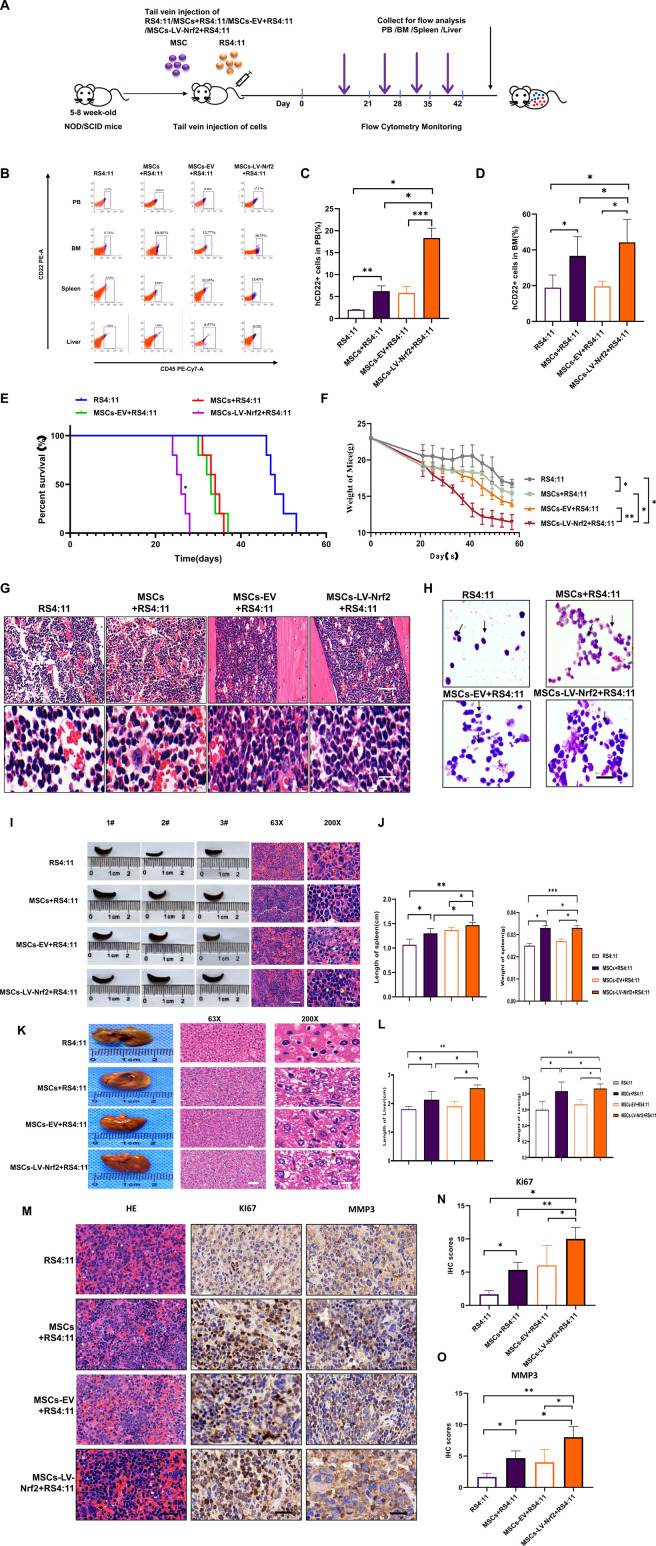
(Continued). Nrf2 upregulation in MSCs enhances B-ALL cell infiltration into extramedullary organs through in vivo experiments. **(A)** Schematic diagram showing NOD-SCID mouse experiment. **(B)** Human ALL primordial cells (CD45+/CD22+) from peripheral blood, bone marrow, liver and spleen of every mouse were shown through flow cytometry. **(C, D)** Histogram showing human CD22+ cell ratio within peripheral blood and myeloid blood of every mouse. **(E)** The curve showing survival changes of mice in each group. **(F)** The mouse weight change curve. **(G)** Myeloid blood H&E staining biopsy of mice. **(H)** Bone marrow smears of bone marrow blood of mice in each group were explored under the ×100 oil immersion objective lens, and the success of model construction was verified by flow cytometry (scale = 10 μ M). **(I, J)** Spleen length of every group and representative splenic H&E staining biopsy from all groups. **(K)** Typical liver H&E staining biopsy in all groups. **(L)** Histogram showing the mouse liver weight and length. **(M)** Representative H&E staining biopsy of spleen and staining sections of MMP3 and KI67 in each group. **(N)** Histogram showing the Ki67 immunohistochemical score. **(O)** Histogram showing the MMP3 immunohistochemical score. All the above experiments were carried out thrice separately.*p < 0.05, **p < 0.01, ***p < 0.001.

According to our previous experiments, overexpression of Nrf2 in MSCs promoted leukemia cell invasion and migration, we successfully constructed B-ALL-PDX mouse models by injecting RS4; 11 (n = 5) and a mixture of RS4; 11 with MSCs/MSCs-EV/MSCs-LV-Nrf2 (4:1) (n = 5) using tail vein injection. More details are displayed in the flow chart (Figure 6A). Four weeks later, mouse peripheral blood samples were collected to detect whether the modeling was successful. At the same time, leukemic cell abundance in bone marrow blood of mice was regularly monitored by flow cytometry (Figure 6B). As a result, human CD22+/CD45+ cell abundance in MSCs-LV-Nrf2+RS4; 11 group remarkably increased relative to RS4; 11 group (Figures 6C, D). PDX mice co-cultured with MSC + RS4; 11 cells had a significantly shorter lifespan especially in MSCs- LV-Nrf2+RS4; 11 group (Figure 6E). During the survival period the MSCs-LV-Nrf2 group showed a significant decrease in body weight (Figure 6F), and the mice were in poor survival condition with pathologic features such as arched backs, curly hair, and anorexia. With regard to bone marrow, aberrant cells were found from the pathological sections of bone marrow in MSCs + RS4; 11, MSCs-EV + RS4; 11 and MSCs-LV-Nrf2+RS4; 11 groups, but less in RS4; 11 group (Figure 6G). The histology of B-ALL mice represented the stage of advanced cancer. Bone marrow smear analysis suggested that leukemia cell number of MSCs-LV-Nrf2+RS4; 11 group markedly elevated (Figure 6H). Compared with the B-ALL-PDX mouse model, the representative H&E staining also showed a more pronounced organ infiltration (Figure 6I), and the trend of infiltration was also evident in representative liver H&E staining (Figure 6G and Supplementary Figure S5). In addition, the spleen length and weight of LV-Nrf2-MSC + RS4; 11 group increased (Figure 6J). The differences in liver weight and length among groups also increased in MSCs-LV-Nrf2+RS4; 11 group (Figures 6K, L). In addition, hematoxylin and eosin (H&E) staining and immunohistochemistry (IHC) were conducted on spleen samples from each group for analyzing Ki-67 and MMP3 expression levels. When MSCs were co-cultured with Nrf2 overexpressed leukemia RS4; 11 cells, B-ALL mice showed the increased Ki67+/MMP3 ratio (Figures 6M–O). This suggests that Nrf2 overexpression in MSCs when co-cultured with leukemia cells promotes leukemia cell infiltration in extramedullary organs by activating the phosphorylation of the SDF-1/CXCR4 signaling axis and its downstream pathway, shortening the survival time of mice.

## 4 Discussion

Interactions between MSCs and leukemia cells have important implications (Dander et al., 2021). Nrf2 accounts for a key transcription factor as well as a regulator for cell antioxidant response. It modulates gene levels encoded by antioxidases and protects the body from various oxidative changes. However, the effect of Nrf2 expression in MSCs within the microenvironment on biological characteristics of leukemia cells remains unclear. This study explore how MSCs interact with B-ALL cells in promoting leukemia cell migration and invasion by regulating Nrf2 expression in MSCs, and further clarify that the overexpression of Nrf2 in MSCs increases leukemia cell infiltration to extramedullary organs, shortens mouse survival time, and provides a powerful experimental basis for developing potential treatments. Based on our findings, Nrf2 and CXCR4 levels in relapsed B-ALL patients significantly increased relative to normal healthy blood donors, which were also confirmed in Nalm-6/RS4; 11 cells. It is suggested that Nrf2 and CXCR4 may have critical effects of B-ALL genesis and progression. Transcriptome sequencing analysis showed that the overexpression of Nrf2 in MSCs enriched its functional expression. Furthermore, GO enrichment analysis revealed a wealth of biological processes, including the expression of “cell genome”. These results further support the regulation of Nrf2 expression in MSCs and enrich a series of pathway cascades that enhance leukemia cell invasion and migration. In our functional experiment, in order to further analyze the relation of Nrf2 expression in MSCs with CXCR4, we co-cultured Nalm-6/RS4; 11 cells with Nrf2 overexpressed/silenced MSCs by lentivirus transfection *in vitro*. Western blotting results showed that Nrf2 regulated the change in CXCR4 expression, which further clarified the positive correlation between CXCR4 and Nrf2. It is suggested that after Nrf2 overexpressed MSCs were co-cultured with Nalm-6/RS4; 11 cells, CXCR4 on Nalm-6/RS4; 11 cell surface can be an important factor activating the SDF-1/CXCR4 axis within the leukemia microenvironment, which further promotes Nrf2 overexpressed MSCs to react with ALL cells (Teicher and Fricker, 2010).

Previous studies also show that, when using the 2D culture model (Gao et al., 2021) to analyze cell migration and invasion ability *in vitro*, compared with Nrf2 silenced MSCs. Nrf2 overexpressed MSCs attract a greater number of leukemia cells to adhere onto their surface through migration and invasion, but the number of adhering leukemia cells significantly decreased by adding AMD 3100. Adding AMD 3100 partially reversed the interaction between Nrf2 overexpressed MSCs and leukemia cells, which suggests that targeting Nrf2 is the potential efficient treatment for reducing leukemia cell infiltration into extramedullary organs and disease progression. Consistent results are found in prior studies on ovarian cancer (Yu et al., 2021). Through suppressing adhesion and promoting mobilization, leukemia cells have changed ability to interact with Nrf2 overexpressed MSCs, thus enhancing therapeutic sensitivity (Fathi and Vietor, 2021). Additionally, *in vivo* experiments revealed that the injection of Nrf2 overexpressed MSCs and RS4; 11 in mice through tail vein significantly increased extramedullary infiltration of RS4; 11 cells and apparently shortened the survival time of mice. Therefore, we suggest that Nrf2 overexpressing MSCs are an important factor in inducing leukemia cell aggressiveness.

According to the KEGG map study, MSCs with Nrf2 overexpression induced the activation of numerous intracellular pathways, such as PI3K-AKT-mTOR, Ras-MAPK, ERK-1-ERK-2 and JAK-STAT pathways. Previous studies have also shown that inhibiting PI3K-AKT and ERK1/2 significantly reduces leukemia cell migration and invasion (Dong et al., 2021). We confirm that MSCs with Nrf2 overexpression induces the phosphorylation in downstream pathways through activating SDF-1/CXCR4 axis, thus enhancing leukemia cell invasion and migration. As a result, SDF-1/CXCR4 axis is a possible target for improving BM-MSCs homing and BM-MSC transplantation therapy, with PI3K-AKT and ERK1/2 being key downstream effectors. After adding PI3K-AKT and ERK1/2 inhibitors, number of leukemia cells migrating and invading declined. In some research, SDF-1/CXCR4-regulated chemotaxis is probably triggered via ERK1/2-activated MAPK(Kodali et al., 2023). The invasive effect is also manifested by the secretion of chemokines and soluble factors to induce tumor cell adhesion and directed chemotaxis (Ragni et al., 2020). Directed chemotaxis is to promote tumor cell adhesion and adjacent tumor cell expression (Waldeck et al., 2020). MSCs protect against tumor cells, which can reduce cancer cell apoptosis rate (Azadniv et al., 2020). In our experimental exploration, chemokines and soluble factors secreted by stromal cells also increased in Nrf2 overexpressed MSCs, thus promoting leukemia cell migration and invasion and promoting infiltration into extramedullary organs.

These results suggest that this effect may be attributed to the fact that Nrf2 overexpressed MSCs promote specific activation of SDF-1/CXCR4 signal axis. In addition, overexpression of Nrf2 in BM-MSCs promotes leukemia cell infiltration into extramedullary organs, which provides a new target for developing effective drugs for the treatment of B-ALL, lays an experimental foundation to explore the possible mechanisms related to leukemia cell growth and invasion, and provides a reference for inhibiting B-ALL infiltration in extramedullary organs.

## 5 Conclusion

**FIGURE 7:**
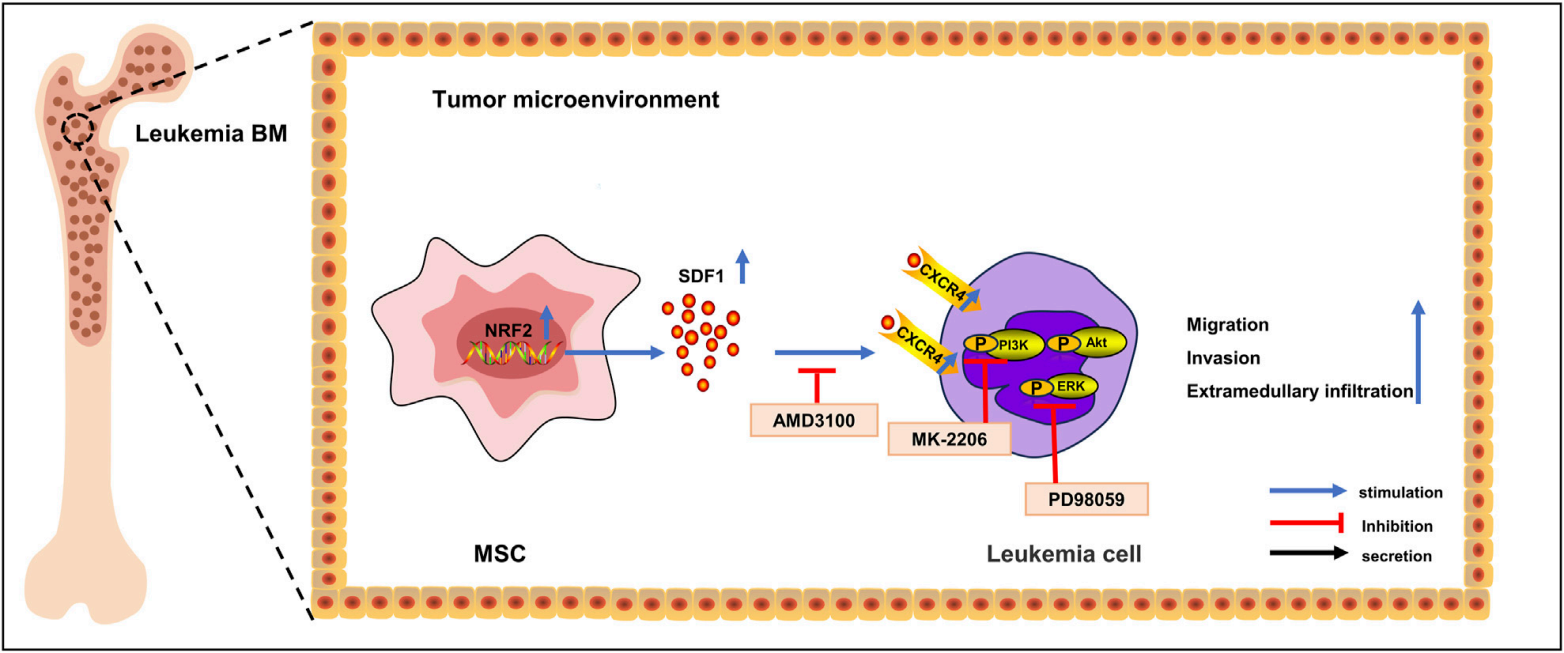
Mechanism diagram. In acute lymphoblastic leukemia cells, Nrf2 overexpression in MSCs in the bone marrow microenvironment promotes the expression of CXCR4 in leukemia cells, which activates the activity of the SDF-1/CXCR4 signaling axis, which in turn activates the activity of its downstream PI3K-AKT/ERK signaling pathway, promotes leukemia cell invasion as well as the infiltration of extramedullary organs, and shortens survival.

These results indicated that Nrf2 overexpressing MSCs promoted specific activation of the SDF-1/CXCR4 signaling axis and triggered downstream phosphorylation of the PI3K/AKT and ERK pathways, which laid an experimental foundation for exploring the possible mechanisms of leukemia cell migration and invasion and provided a reference to inhibit the infiltration of B-ALL into extramedullary organs (Figure 7).

## Data Availability

The data presented in the study are deposited in the https://ngdc.cncb.ac.cn/gsa-human repository, accession number GSAHuman: HRA005503.

## Abbreviations

B-ALL, B-cell acute lymphoblastic leukemia; BM-MSC, Bone Marrow Mesenchymal Stem Cells; BM, Myeloid Blood; BMM, Bone marrow microenvironment; CXCR4:Chemokine receptor 4; DMSO, dimethyl sulfoxide; EMT, Epithelial Mesenchymal Transformation; GEO:Gene expression omnibus; GO:Gene ontology; GSEA:Gene set enrichment analysis; IHC, Immunohistochemistry; IF, Immunofluorescence Staining; MSCs, Mesenchymal Stem Cells; mRNA, Messenger RNA; PB, Peripheral Blood; qRT-PCR, Real-Time Quantitative Reverse Transcription PCR; RNA-seq, Ribonucleic acid sequencing; TME:Tumor microenvironment; NOD/SCID:Nonobese diabetes/severe combined immunodeficiency.
