# Correction: Overexpression of Nrf2 in bone marrow mesenchymal stem cells promotes B-cell acute lymphoblastic leukemia cells invasion and extramedullary organ infiltration through stimulation of the SDF-1/CXCR4 axis

**DOI:** 10.3389/fphar.2025.1660169

**Published:** 2025-08-08

**Authors:** Lin Zheng, Chengyun Pan, Dan Ma, Qin Shang, Tianzhen Hu, Tianzhuo Zhang, Qian Kang, Xiuying Hu, Shuyun Cao, Li Wang, Hong Luo, Jishi Wang

**Affiliations:** ^1^ Department of Hematology, Affiliated Hospital of Guizhou Medical University, Guiyang, China; ^2^ Department of Clinical Medical School, Guizhou Medical University, Guiyang, China; ^3^ Department of Guizhou Province Hematopoietic Stem Cell Transplantation Center and Key Laboratory of Hematological Disease Diagnostic and Treatment Centre, Guiyang, China; ^4^ Guizhou Provincial Engineering Technology Research Center for Chemical Drug R&D, Guizhou Medical University, Guiyang, China

**Keywords:** B-acute lymphocytic leukemia, MSCs, Nrf2, SDF-1/CXCR4, extramedullary infiltration

In the published article, there was an error in Figures 2A, C, Figures 3N, O, Figures 5A, C, as published. The error was caused by an image misalignment during image selection of raw data. The corrected Figures 2A, C, Figures 3N, O, Figures 5A, C and their captions appear below.

**FIGURE 2:**
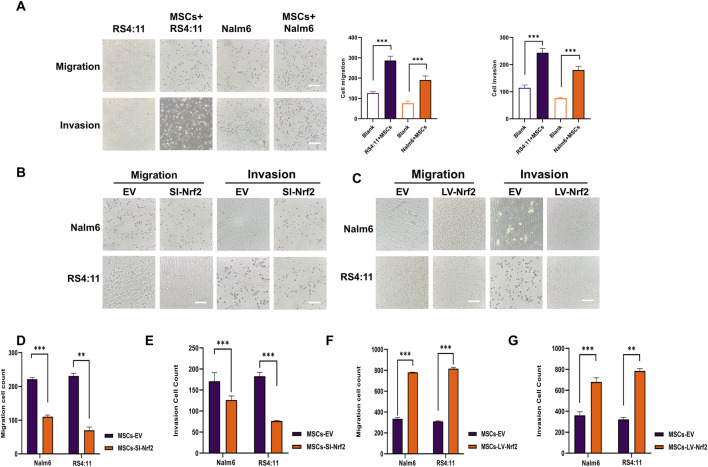
Nrf2 in BMSCs promotes B-ALL cell migration and invasion **(A)** Transwell assays performed for analyzing cell invasion and migration after incubation with Nalm-6/RS4; 11 alone and co-culture with MSCs for 24 h **(B, D, E)** Downregulation of Nrf2 expression in MSCs was tested for migration and invasion rates of Nalm6/RS4:11 cells in the microcellular compartment at 24 h. Migration and invasion rates were significantly decreased in the downregulated group (p < 0.01). **(C,F,G)** Upregulation of Nrf2 expression in MSCs revealed a significant increase in the migration and invasion rate of Nalm6/RS4: 11 in cells within the upregulated group at 24 h. Each assay was conducted thrice independently and denoted as mean ± SD. *p < 0.05, **p < 0.01, ***p < 0.001.

**FIGURE 3:**
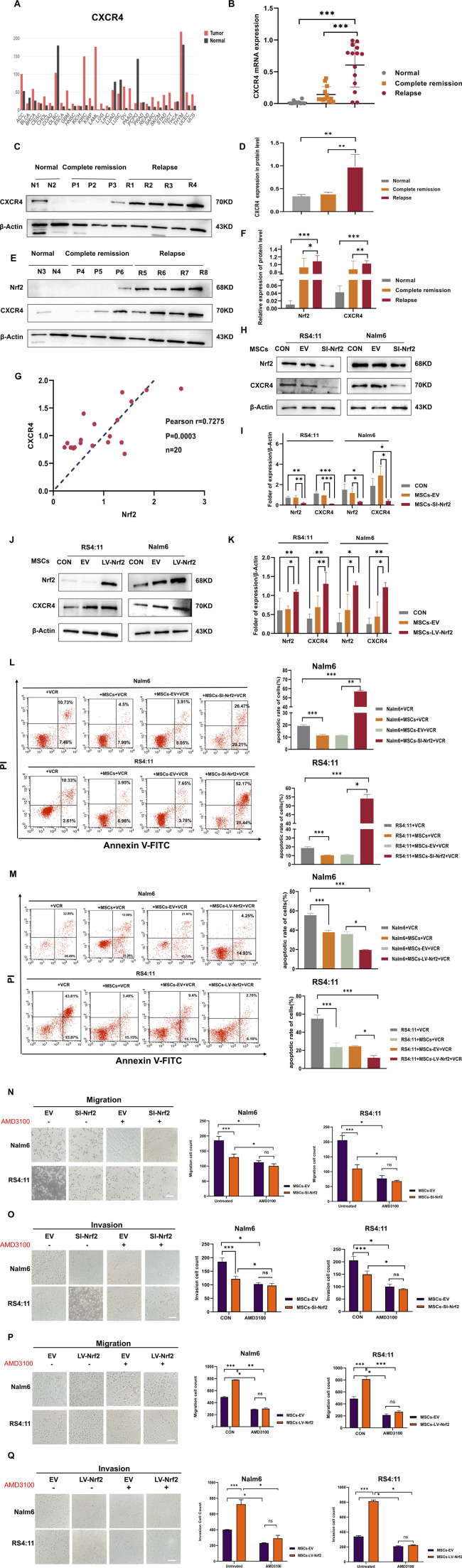
CXCR4 overexpression in B-ALL cells was positively related to Nrf2 **(A)** GEPIA database analysis for CXCR4 levels within tumor and non-carcinoma tissues. **(B)** RT-PCR assay on CXCR4 levels within healthy (n = 10), complete remission (n = 20) and relapsed (n = 20) B-ALL patients. **(C,D,E,F)** Western blotting was conducted for detecting CXCR4 and Nrf2 protein levels among healthy (n = 4), complete remission (n = 6) and relapsed B-ALL patient (n = 8) samples. **(G)** Association of Nrf2 with CXCR4 in B-ALL was measured through RT-PCR (n = 20, r = 0.7275 = 0.5232, p = 0.0003). **(H,I)** Western blotting was performed to detect the expression of CXCR4 in RS4; 11/Nalm-6 in the co-culture system after downregulation of Nrf2 in MSCs. **(J,K)** Western blotting was performed to detect the expression of CXCR4 in RS4; 11/Nalm-6 in the co-culture system after upregulation of Nrf2 in MSCs. **(L)** MSCs-SI-Nrf2 significantly affected leukemia cell sensitivity to vincristine. **(M)** MSCs-LV-Nrf2 had a similarly significant change in changing leukemia cell sensitivity to vincristine. **(N–Q)** Transwell tests were performed to detect the number of Nalm-6 and RS4; 11 cells migrating and invading in the MSCs-EV, MSCs-SI-Nrf2, MSCs-LV-Nrf2, and treated AMD3100 (20 μM, upper chamber) groups, respectively, after 24 h of cell incubation. Each assay was conducted thrice independently and denoted as mean ± SD. *p < 0.05, **p < 0.01, ***p < 0.001.

**FIGURE 5:**
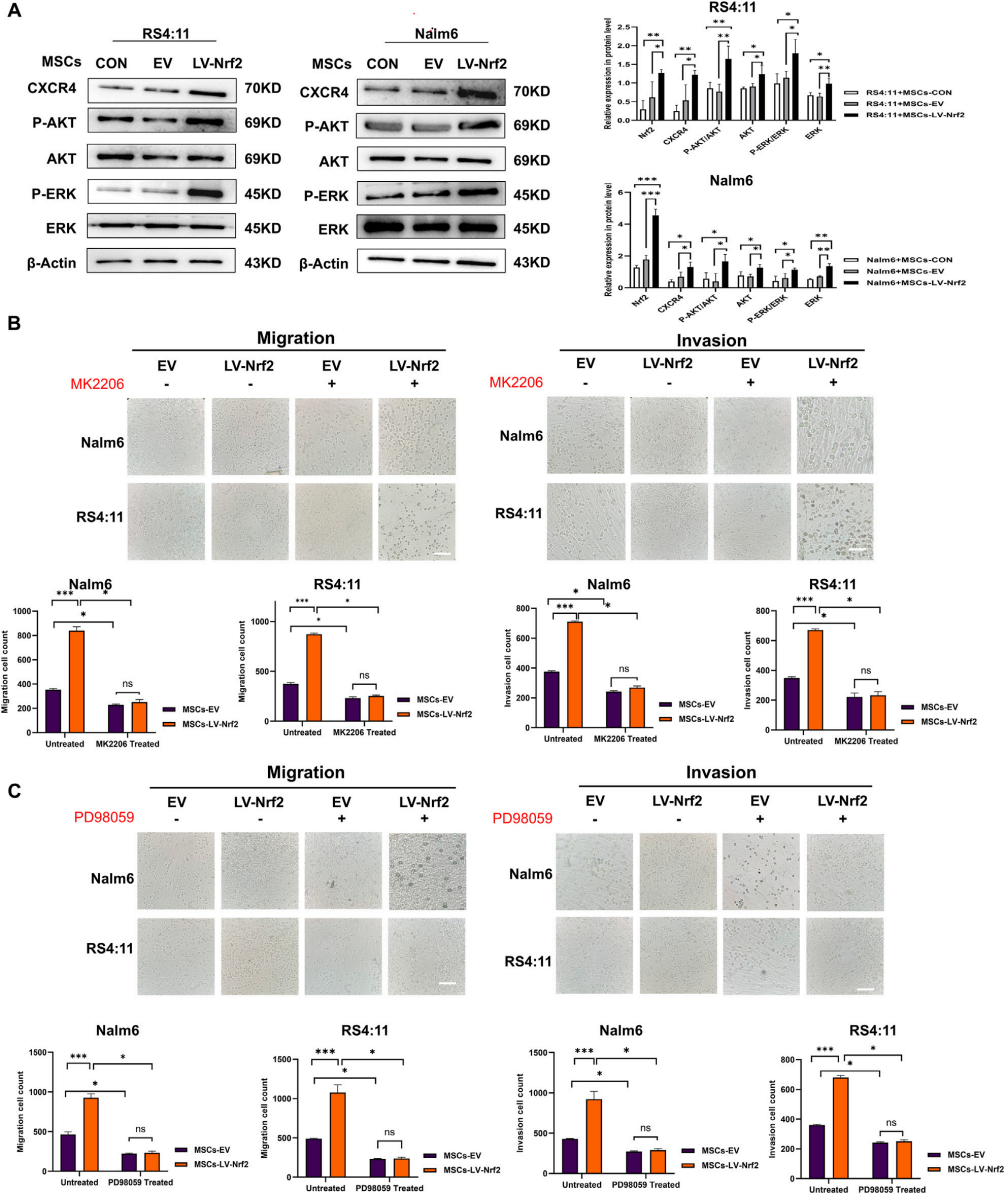
PI3K/AKT and ERK pathway activation mediated by Nrf2 overexpression in MSCs made a vital impact on B-ALL cell invasion and migration. **(A)** PI3K/AKT and ERK pathway in MSCs-LV-Nrf2 and RS4; 11/Nalm-6 co-culture conditions was evaluated through Western blotting. Histogram showed protein level quantification. **(B)** Addition of AKT inhibitor (MK2206), the results showed that the migration and invasion ability of leukemia cells were attenuated, and there was no statistically significant difference between the EV and LV-Nrf2 groups. **(C)** Addition of ERK inhibitor (PD98059) to the upper compartment also significantly blocked the migration and invasion ability of leukemia cells, with no statistically significant difference between the EV and LV-Nrf2 groups. Each assay was conducted thrice independently and represented by average soil SD. *p < 0.05, **p < 0.01, ***p < 0.001.

The original version of this article has been updated.

