# Supplementary material for: Overexpression of Nrf2 in bone marrow mesenchymal stem cells promotes B-cell acute lymphoblastic leukemia cells invasion and extramedullary organ infiltration through stimulation of the SDF-1/CXCR4 axis
Source: Front Pharmacol. 2024 Jul 16;15:1393482. doi: 10.3389/fphar.2024.1393482 (PMC11286583; doi:10.3389/fphar.2024.1393482)
Supplement: Supplementary file 1 [file DataSheet1.docx]

**Additional materials and methods**

## RNA sequencing: RNA extraction

Trizol reagent (Invitrogen) was utilized to extract total RNA from Nrf2 overexpressed MSCs and MSCs-EV samples. Then, the Bioanalyzer 2200 (Aligent) was utilized to check RNA quality, which was later preserved under -80 °C. RNA of RIN > 8.0 was suitable for rRNA depletion.

**RNA sequencing: cDNA library construction**

In line with specific protocols, Ion Total RNA-Seq Kit v2.0 (Life Technologies) was utilized to prepare a cDNA library for every collected RNA sample to conduct single-ended sequencing. In general, this scheme consisted of the use of bivalent cations at 94 °C to deplete rRNA and the fragmentation of it into 150,200 bp. The cut RNA fragment was later prepared to first strand cDNA through reverse transcription, then, the second strand cDNA was prepared, and the end of the fragment was repaired. Additionally, a tail was added and connected with the index adapter. The target bands were harvested by nucleic acid binding beads. PCR was conducted for product purification and enrichment to produce the final cDNA library, which was quantified with Agilent 2200.

## RNA sequencing

The cDNA library was processed according to the commercially available scheme for proton sequencing. Following sample dilution and mixing, the mixed sample was processed with an OneTouch 2 instrument (Life Technologies) prior to enrichment onto an OneTouch 2ES station (Life Technologies). Using the Ion PI Template OT2 200 Kit v2.0 (Life Technologies), the sample was then used for preparing the template positive Ion PI "Ion Sphere" particles (Life Technologies). Later, the template was enriched, loaded on IP1v2 proton chip (Life Technologies), and sequenced on a proton sequencer according to NovelBio Corp. Ion PI Sequencing 200Kit v2.0 (Life Technologies) instructions.

## RNA sequencing: quality control

By removing the linking subsequence, a clean reading segment was obtained from the original reading segment, a reading segment of > 5% fuzzy bases (marked as N) as well as a low-quality reading segment that contained > 20% bases, whose mass was less than 13.

## Bioinformatics analysis: gene expression analysis

The genes were counted by HTseq, and later gene expression was standardized using the RPKM Method.

## **Bioinformatics analysis: differentially expressed gene (DEG) identification.**

DEGs were obtained with the DEseq algorithm. According to differential gene analysis results, volcano maps were plotted using Chipster, cluster heatmaps were plotted using the R package gplots, and colors were determined based on the screening criteria.

**
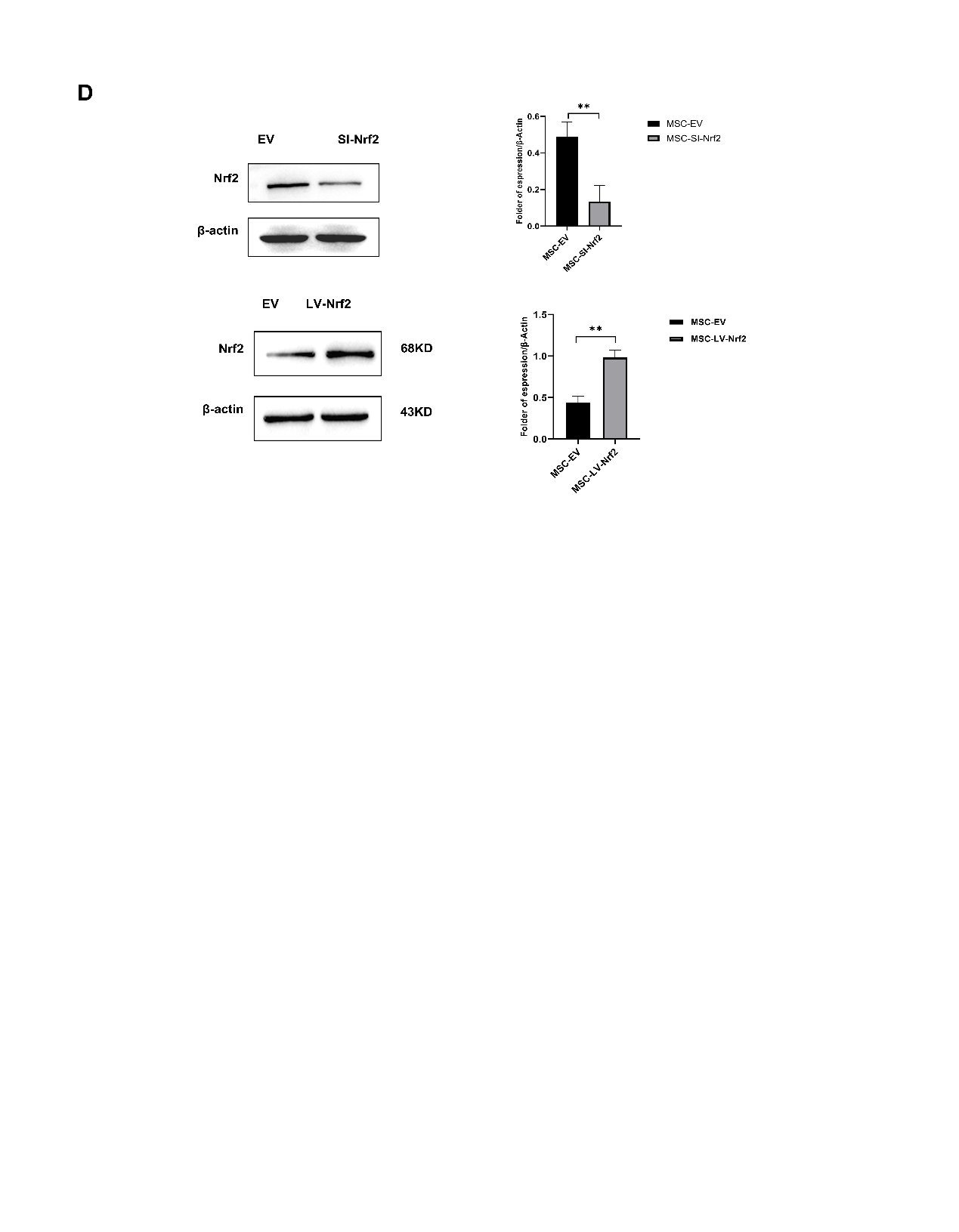

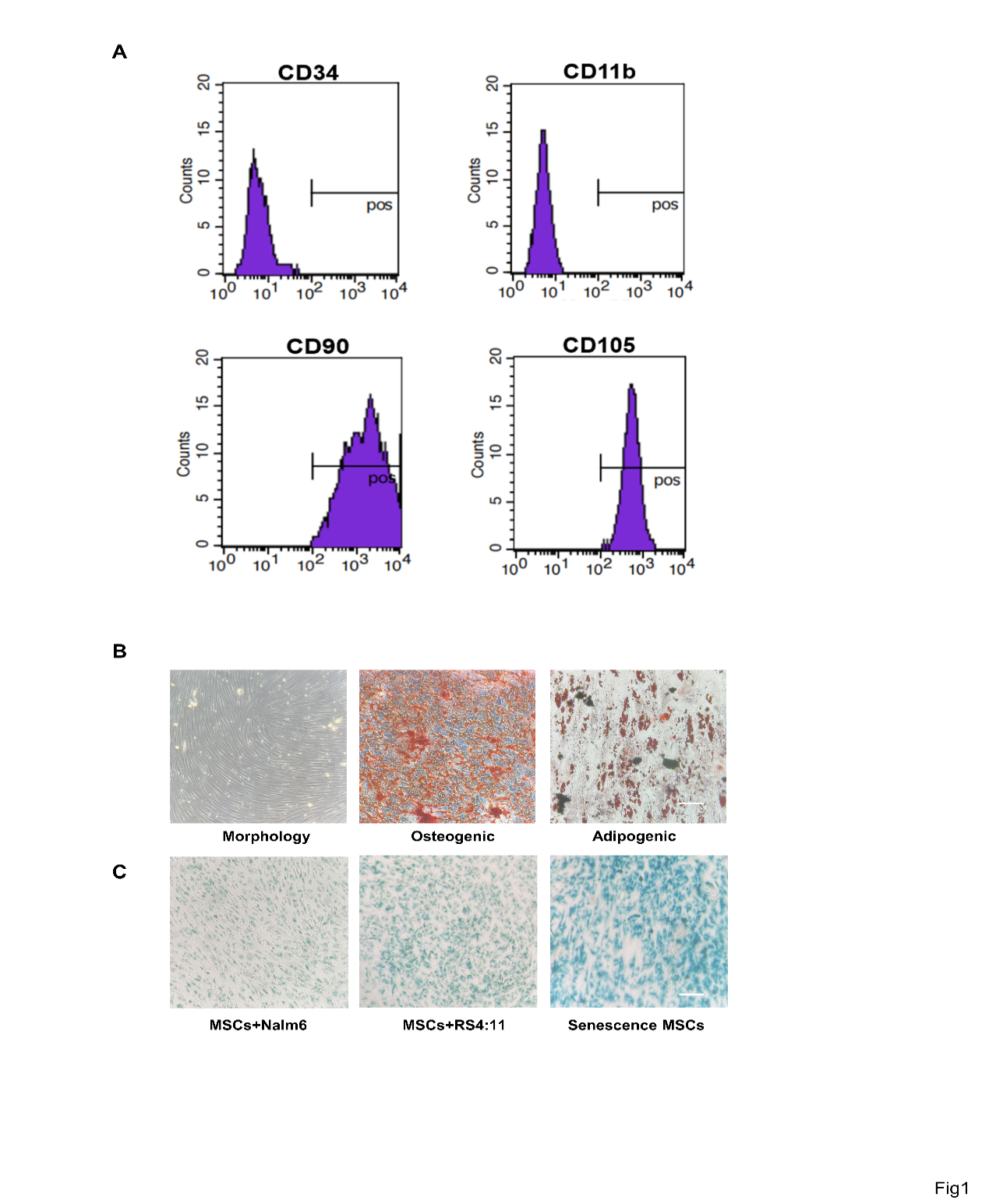
Additional figures**

**
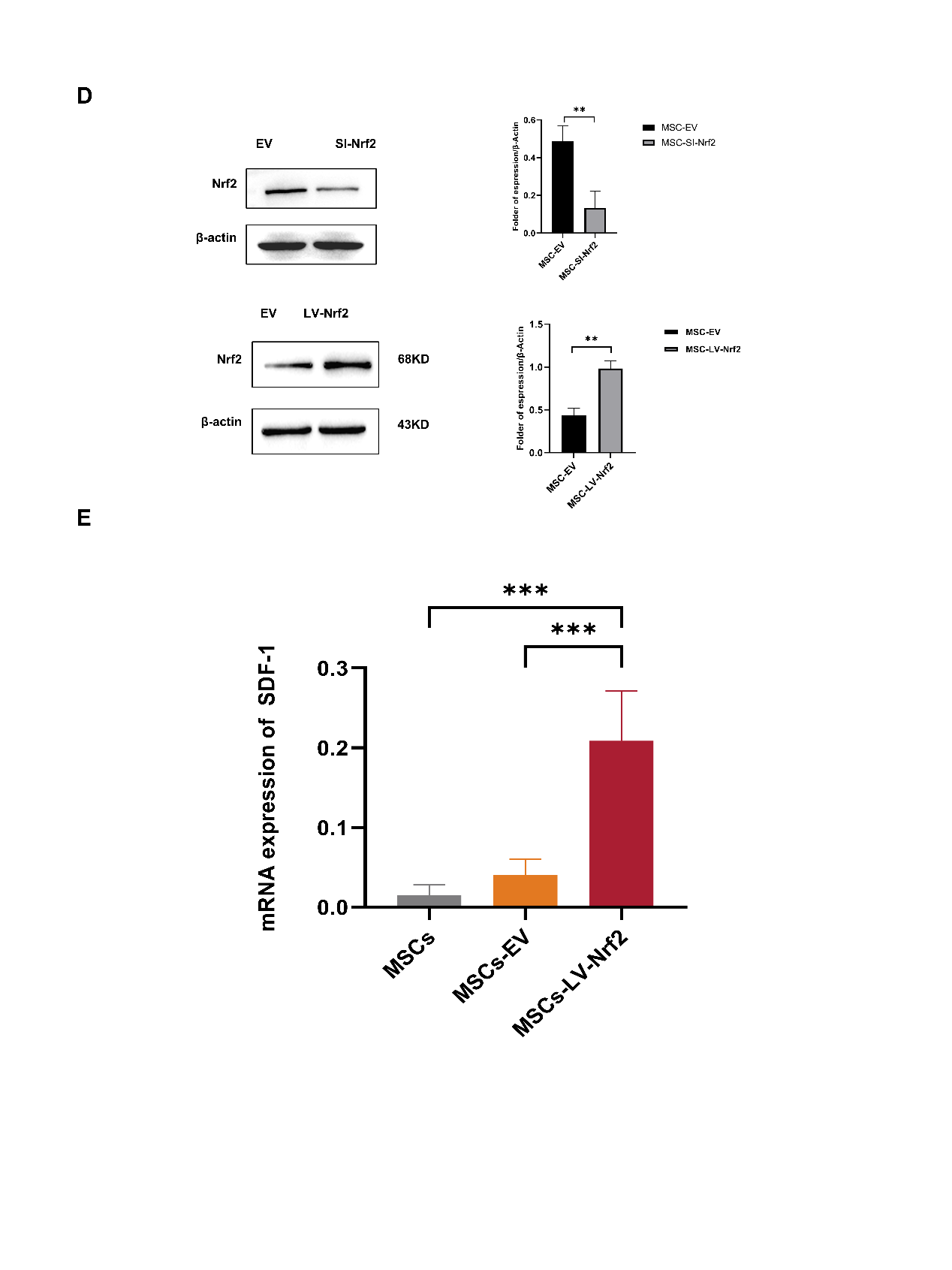
**

**Fig S1. Mesenchymal stem cell characterization.**

（**A**）MSCs CD105 and CD90 were positive, CD34 and CD11b were negative. These standards are used to define marrow mesenchymal stem cells. In this experimental design, we confirmed and identified by flow cytometry at the early stage. (**B**) Nevertheless, 72-h col-culture of MSCs and Nalm-6/RS4;11 after MSCs were exposed to lipogenic and osteogenic differentiation assays, the co-cultured MSCs had diminished differentiation ability relative to MSCs cultured alone(200× , scale bars, 50 µm). (**C**)The changes in cellular senescence of both groups could be found from senescence-related β-galactosidase staining, and the co-cultured MSCs had markedly increased senescence degree compared with mono-cultured MSCs (200 ×, scale bar, 50 µm). It is suggested that MSCs in the leukemic microenvironment are differentially attenuated in terms of their differentiation ability and undergo senescence changes under co-culture conditions. **(D)** Protein levels of Nrf2 in MSCs of the blank group (EV), Nrf2 down-regulated group (SI-Nrf2), and Nrf2 up-regulated group (LV-Nrf2), mean ± SD deviation.

**
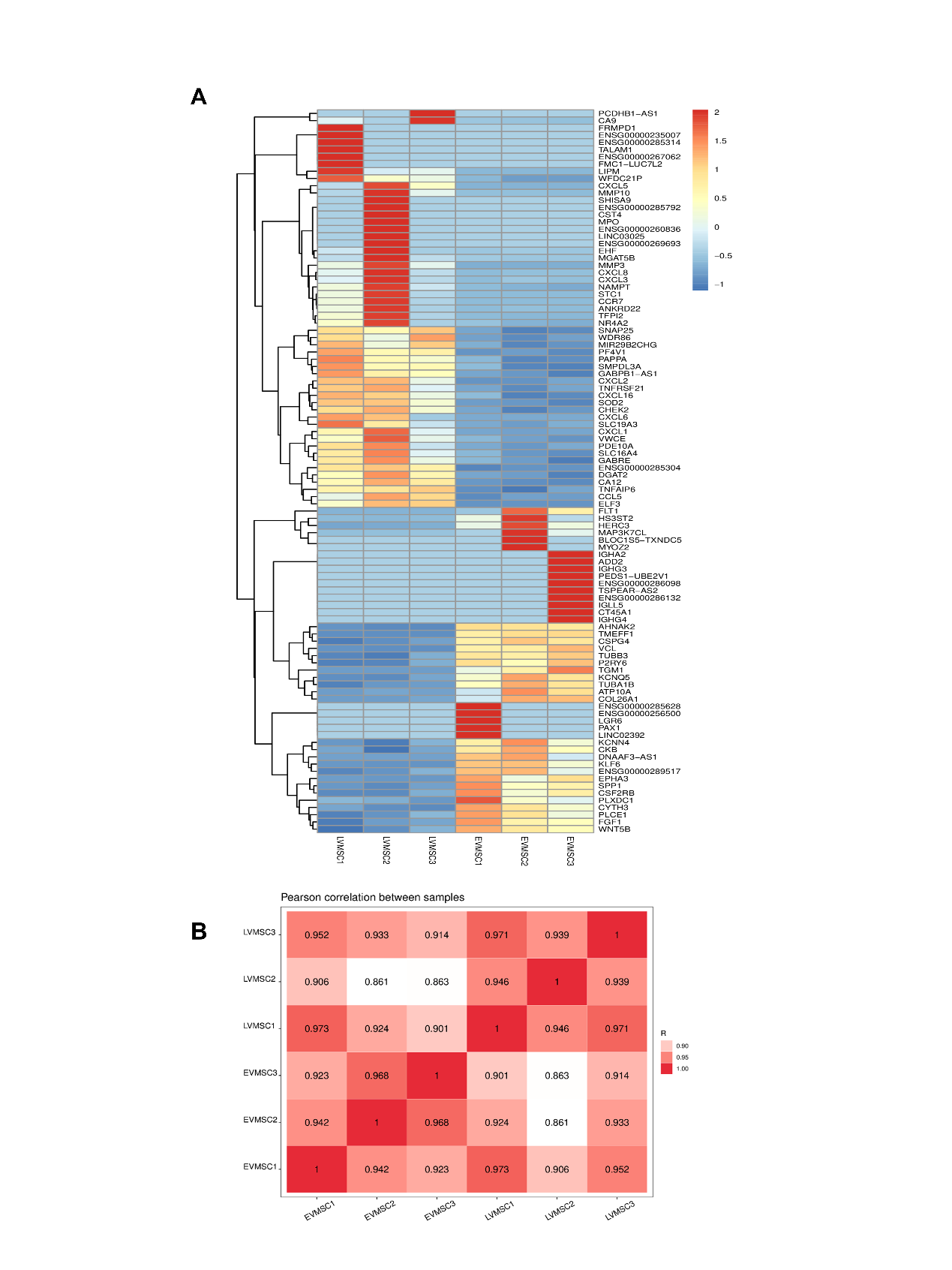
**

**
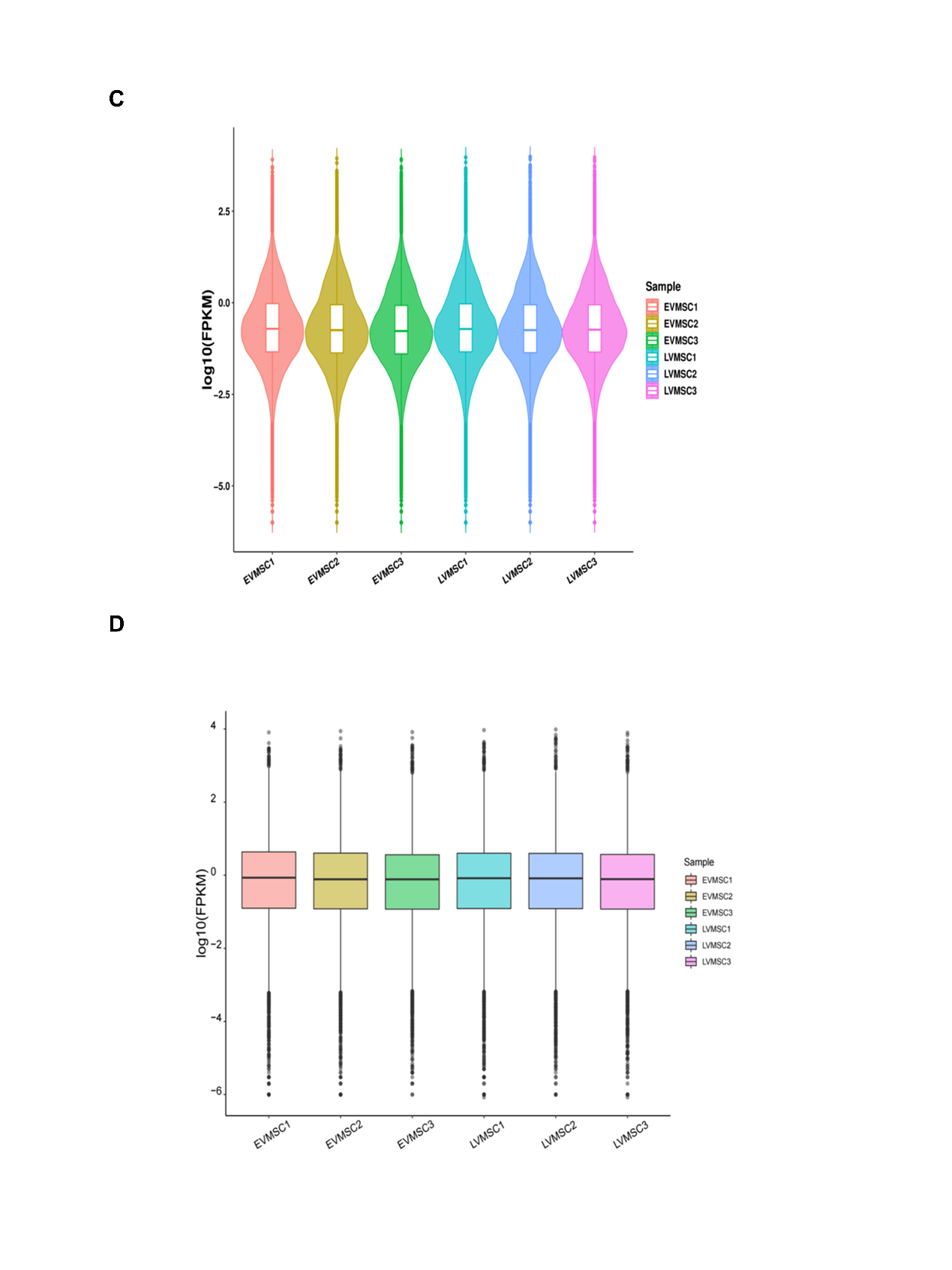
**

**
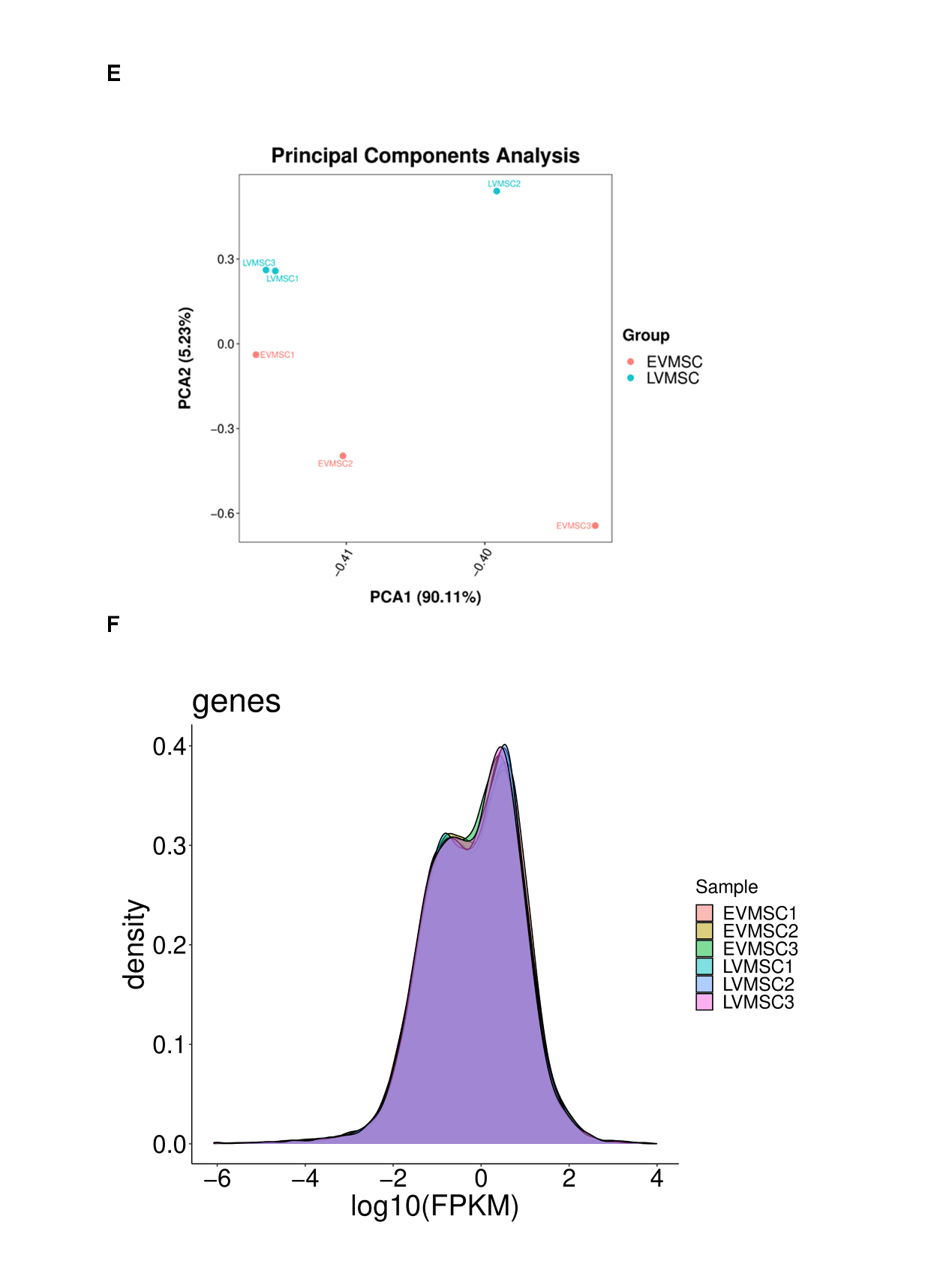
**

**Fig S2.** **Biological functions enriched by RNA-Seq in LV-Nrf2-MSCs**

（**A**）Heatmap of expression levels in the data. Blue color indicates low expression and red color indicates high expression. (**B**)MSCs-LV-Nrf2 and control MSCs were separated by principal components, and sample correlation analysis confirmed the greater variability between groups than within groups. (**B, C**) Violin and box plots of the expression of MSCs-LV-Nrf2 samples via control and experimental groups. (**D**) The gene expression density of MSCs-EV and MSCs-LV-Nrf2. (**E**) Principal component analysis based on the control (MSCs-EV) and experimental (MSCs-LV-Nrf2) groups showed that the variables in the control and MSCs-LV-Nrf2 groups were significantly different from each other.


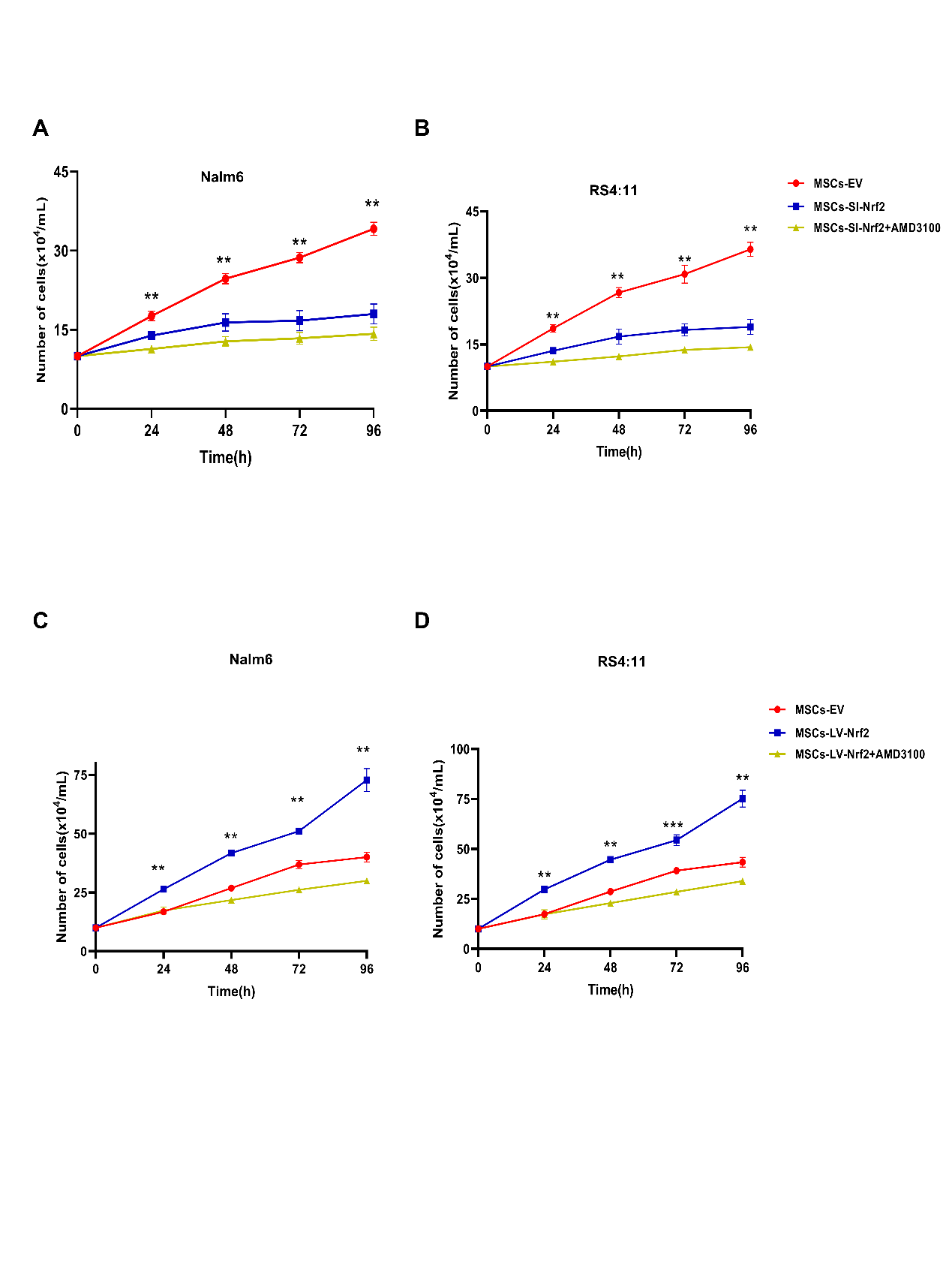


**Fig S3. Nrf2 overexpression in mesenchymal stem cells promotes B-ALL cell proliferation.**

(**A,B**) Interestingly, Nalm6/RS4:11 proliferation counts were detected by co-culturing with MSCs-EV, MSCs-SI-Nrf2, and MSCs-SI-Nrf2 + AMD3100 (20 μM) groups for 72 h after 24 h, 48 h, 72 h, and 96 h of cell incubation. Mean ± SEM, n = 4. (**C, D**) Cell proliferation numbers for Nalm6 and RS4:11. After cells were incubated for 24 h, 48 h, 72 h and 96 h, co-cultured with MSCs-EV, MSCs-LV-Nrf2 and MSCs-LV-Nrf2 + AMD3100(CXCR4 specific inhibitors) (20 μM) groups for 72 h. "*" in the graph indicates the significant difference between MSCs-LV- Nrf2 and MSCs-LV-Nrf2 + AMD3100 groups were significantly different from each other. *p < 0.05, **p < 0.01, ***p < 0.001.


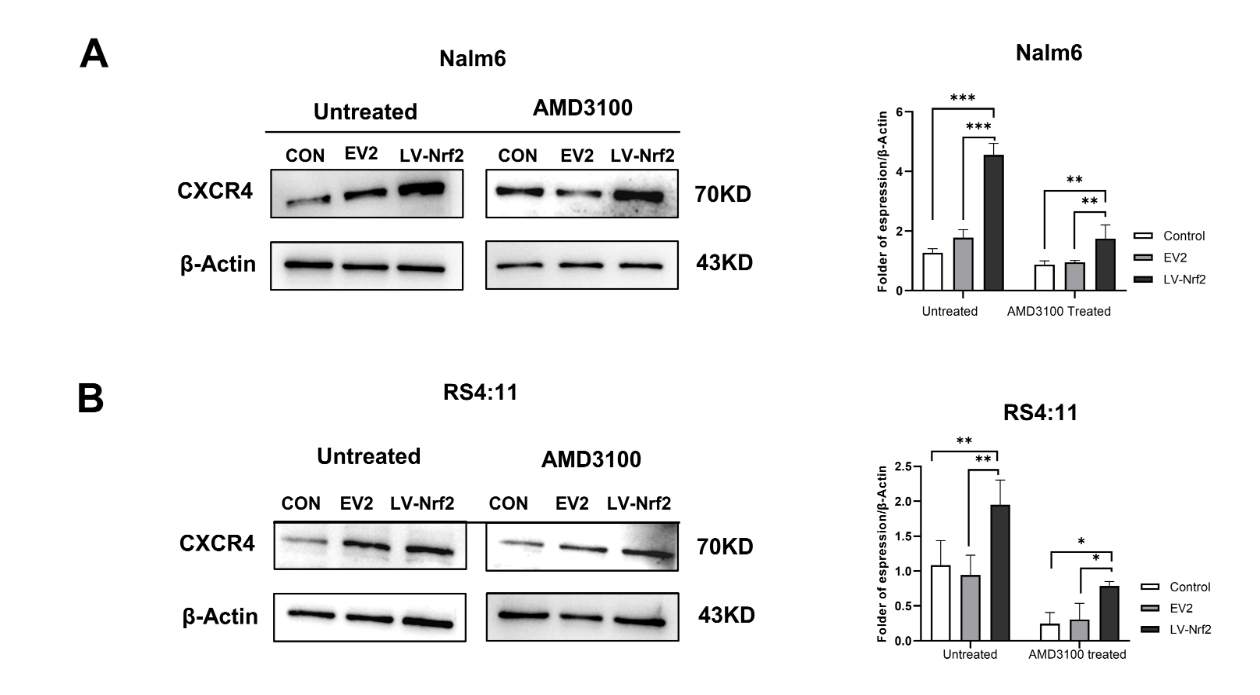


**Fig S4. Results of protein immunoblotting experiments showing protein expression levels of CXCR4 after addition of AMD3100.**

(**A, B**) Nalm6/RS4:11 were co-cultured with MSCs-CON,MSCs-EV,MSCs-LV-Nrf2 groups for 72h, respectively, and then treated with AMD3100 to detect Nrf2 expression in B-ALL cells by Western blotting. The relative gray values were shown in histogram. Statistical differences were determined using the Student's t-test. *p<0.05, **p<0.01, ***p<0.001.


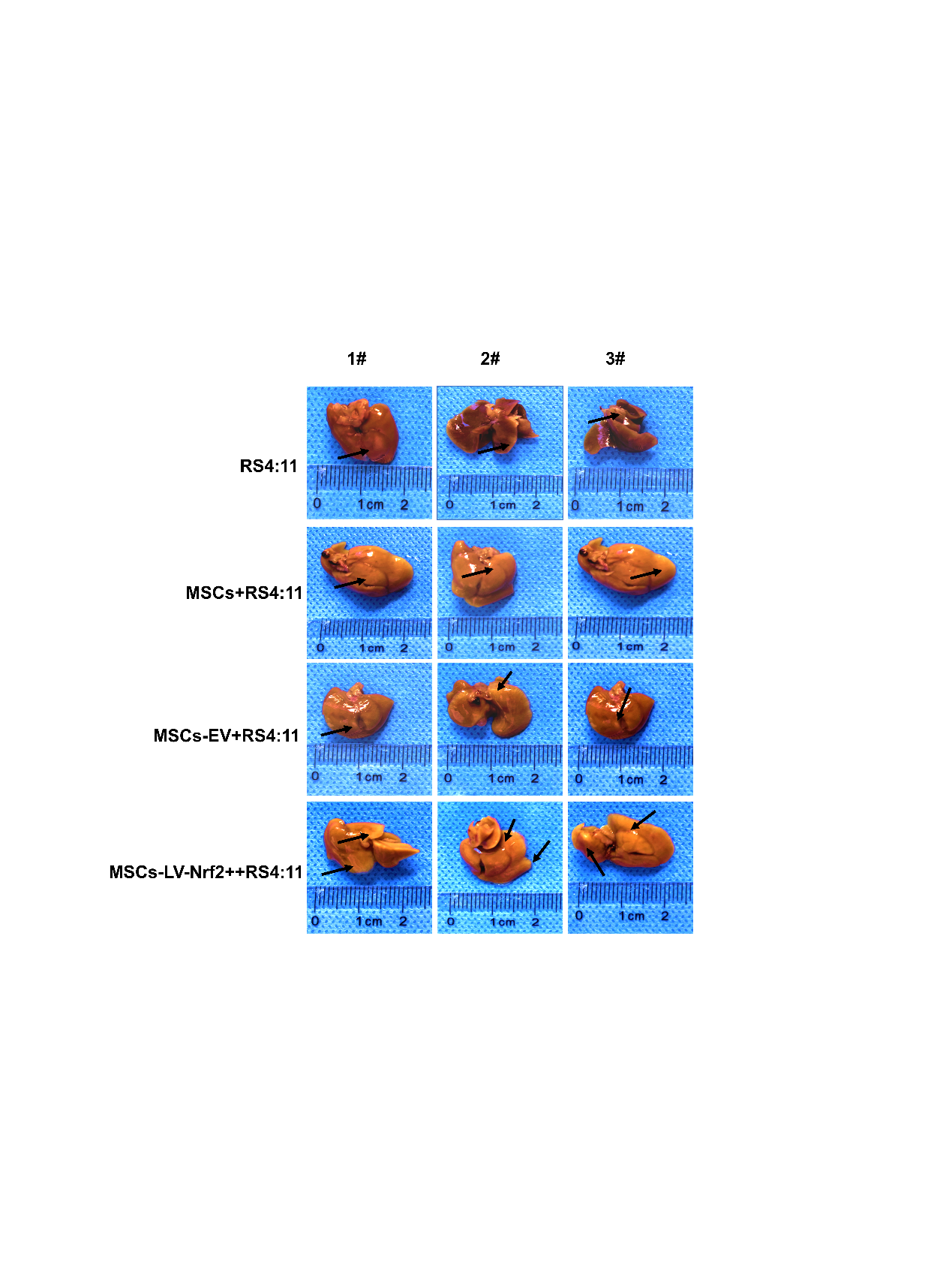
**Fig S5.** **Morphological changes in liver after extramedullary infiltration.**

The appearance of mouse livers had obvious foci of leukemic cell infiltration, with curly and irregular liver morphology, and the infiltration was more obvious in the MSCs-LV-Nrf2+RS4:11 group.

**Additional tables**

**Table S1. Characteristics of patient samples.**

| Samples | Age | Sex | Disease status | WBC (10^9^/L) | Hb  (g/l) | PLT  (10^9^/L) | %Blasts (BM) |
| --- | --- | --- | --- | --- | --- | --- | --- |
| B-ALL1 | 26 | M | complete remission | 7.68 | 67 | 55 | 2.15 |
| B-ALL2 | 23 | M | complete remission | 6.57 | 121 | 70 | 3.26 |
| B-ALL3 | 27 | M | complete remission | 13.26 | 69 | 35 | 1.01 |
| B-ALL4 | 21 | M | complete remission | 8.54 | 57 | 41 | 2.67 |
| B-ALL5 | 18 | F | complete remission | 7.75 | 55 | 30 | 1.25 |
| B-ALL6 | 19 | F | complete remission | 7.77 | 58 | 150 | 0.87 |
| B-ALL7 | 23 | F | complete remission | 9.13 | 69 | 50 | 0.87 |
| B-ALL8 | 23 | F | complete remission | 8.17 | 71 | 114 | 1.17 |
| B-ALL9 | 25 | F | complete remission | 8.19 | 87 | 71 | 0.78 |
| B-ALL10 | 31 | M | complete remission | 8.88 | 85 | 54 | 1.27 |
| B-ALL11 | 33 | M | complete remission | 6.57 | 89 | 27 | 2.76 |
| B-ALL12 | 24 | M | complete remission | 7.19 | 97 | 78 | 2.01 |
| B-ALL13 | 44 | M | complete remission | 10.31 | 90 | 26 | 2.57 |
| B-ALL14 | 41 | M | complete remission | 17.13 | 93 | 77 | 1.88 |
| B-ALL15 | 32 | F | complete remission | 13.57 | 91 | 23 | 1.06 |
| B-ALL16 | 23 | F | complete remission | 6.58 | 67 | 51 | 2.89 |
| B-ALL17 | 21 | F | complete remission | 7.13 | 79 | 53 | 1.17 |
| B-ALL18 | 27 | F | complete remission | 7.58 | 78 | 79 | 2.27 |
| B-ALL19 | 37 | F | complete remission | 7.59 | 73 | 73 | 0.19 |
| B-ALL20 | 36 | F | complete remission | 8.76 | 89 | 26 | 0.96 |
| B-ALL21 | 35 | M | complete remission | 8.53 | 88 | 23 | 3.21 |
| B-ALL22 | 31 | M | complete remission | 8.35 | 87 | 25 | 2.67 |
| B-ALL23 | 51 | M | complete remission | 10.35 | 57 | 73 | 3.19 |
| B-ALL24 | 33 | M | complete remission | 10.56 | 110 | 57 | 3.11 |
| B-ALL25 | 43 | F | complete remission | 7.98 | 54 | 59 | 2.71 |
| B-ALL26 | 41 | F | complete remission | 6.57 | 78 | 71 | 0.98 |
| B-ALL27 | 37 | F | complete remission | 8.15 | 108 | 79 | 3.10 |
| B-ALL28 | 31 | F | complete remission | 8.97 | 67 | 32 | 3.87 |
| B-ALL29 | 33 | F | complete remission | 9.13 | 77 | 37 | 0.61 |
| B-ALL30 | 35 | F | complete remission | 9.15 | 73 | 29 | 0.93 |
| B-ALL31 | 41 | F | complete remission | 6.53 | 78 | 28 | 0.19 |
| B-ALL32 | 25 | F | complete remission | 5.78 | 81 | 33 | 3.26 |
| B-ALL33 | 38 | M | complete remission | 5.88 | 89 | 47 | 3.92 |
| B-ALL34 | 41 | M | complete remission | 9.17 | 93 | 46 | 2.08 |
| B-ALL35 | 40 | M | complete remission | 15.17 | 95 | 53 | 2.93 |
| B-ALL36 | 43 | M | complete remission | 16.01 | 58 | 121 | 1.78 |
| B-ALL37 | 45 | M | complete remission | 13.89 | 57 | 115 | 3.29 |
| B-ALL38 | 37 | M | complete remission | 14.21 | 69 | 77 | 1.93 |
| B-ALL39 | 40 | M | complete remission | 13.53 | 73 | 56 | 0.56 |
| B-ALL40 | 22 | M | complete remission | 12.17 | 68 | 45 | 3.27 |
| B-ALL41 | 25 | M | complete remission | 8.19 | 60 | 28 | 1.08 |
| B-ALL42 | 35 | F | complete remission | 7.19 | 58 | 21 | 1.79 |
| B-ALL43 | 33 | F | relapse | 6.90 | 78 | 36 | 41.57 |
| B-ALL44 | 37 | F | relapse | 5.67 | 88 | 37 | 45.37 |
| B-ALL45 | 32 | F | relapse | 5.31 | 87 | 45 | 51.78 |
| B-ALL46 | 35 | F | relapse | 7.57 | 93 | 44 | 60.91 |
| B-ALL47 | 31 | F | relapse | 8.02 | 89 | 78 | 71.37 |
| B-ALL48 | 27 | F | relapse | 8.19 | 93 | 51 | 38.01 |
| B-ALL49 | 26 | F | relapse | 9.04 | 69 | 57 | 36.57 |
| B-ALL50 | 25 | M | relapse | 9.17 | 76 | 26 | 27.95 |
| B-ALL51 | 28 | M | relapse | 8.56 | 73 | 31 | 67.82 |
| B-ALL52 | 35 | M | relapse | 8.89 | 135 | 37 | 57.39 |
| B-ALL53 | 37 | M | relapse | 13.71 | 58 | 26 | 27.76 |
| B-ALL54 | 36 | M | relapse | 4.53 | 121 | 43 | 39.01 |
| B-ALL55 | 37 | M | relapse | 4.51 | 106 | 34 | 55.73 |
| B-ALL56 | 38 | F | relapse | 4.44 | 57 | 52 | 29.56 |
| B-ALL57 | 31 | F | relapse | 5.71 | 55 | 51 | 22.04 |
| B-ALL58 | 35 | F | relapse | 5.70 | 56 | 61 | 31.17 |
| B-ALL59 | 28 | F | relapse | 5.89 | 90 | 65 | 88.01 |
| B-ALL60 | 26 | F | relapse | 6.04 | 91 | 53 | 39.76 |
| B-ALL61 | 28 | F | relapse | 6.67 | 67 | 51 | 37.47 |
| B-ALL62 | 29 | F | relapse | 6.63 | 61 | 55 | 43.19 |
| B-ALL63 | 27 | M | relapse | 8.71 | 78 | 57 | 52.67 |
| B-ALL64 | 23 | M | relapse | 3.97 | 73 | 67 | 37.56 |
| B-ALL65 | 32 | M | relapse | 6.78 | 75 | 61 | 33.42 |
| B-ALL66 | 33 | M | relapse | 3.56 | 79 | 63 | 39.08 |
| B-ALL67 | 31 | M | relapse | 4.55 | 65 | 71 | 27.18 |
| B-ALL68 | 37 | M | relapse | 5.73 | 83 | 77 | 44.87 |
| B-ALL69 | 38 | M | relapse | 6.08 | 81 | 68 | 45.91 |
| B-ALL70 | 35 | M | relapse | 13.98 | 67 | 101 | 43.71 |
| B-ALL71 | 33 | F | relapse | 19.76 | 79 | 25 | 37.92 |
| B-ALL72 | 37 | F | relapse | 16.90 | 77 | 17 | 27.73 |
| B-ALL73 | 41 | F | relapse | 12.42 | 73 | 28 | 81.03 |
| B-ALL74 | 35 | F | relapse | 9.03 | 59 | 16 | 52.08 |
| B-ALL75 | 41 | F | relapse | 5.56 | 87 | 17 | 57.16 |
| B-ALL76 | 31 | F | relapse | 3.08 | 81 | 23 | 31.04 |
| B-ALL77 | 27 | M | relapse | 3.27 | 106 | 51 | 29.48 |
| B-ALL78 | 29 | M | relapse | 3.58 | 103 | 57 | 37.91 |
| B-ALL79 | 25 | M | relapse | 4.07 | 78 | 107 | 35.01 |
| B-ALL80 | 19 | M | relapse | 5.57 | 93 | 41 | 47.88 |

Abbreviation: M: male; F: female; ALL: Acute Lymphoblastic Leukemia; WBC: white blood cell; Hb: hemoglobin; PLT: platelets; BM: Bone Marrow.

**Table S2. Antibodies used for flow cytometry.**

| Marker | Fluorochrome | Source | Identifier |
| --- | --- | --- | --- |
| CD11b | APC | Beckman Coulter | Cat# 336707 |
| CD45 | PE-Cy7 | Beckman Coulter | Cat# IM3548 |
| CD90 | APC | BD Pharmingen | Cat# 565551 |
| CD105 | APC | BD Pharmingen | Cat# 517926 |
| CD34 | APC | Biolegend | Cat# 328318 |
| CD22 | APC | Biolegend | Cat# 8031008 |

**Table S3.** **The characteristics of the primers used for qRT-PCR.**

| GENE | Primer | Sequence (5′->3′) |
| --- | --- | --- |
| β-Actin | Forward Primer | CTACCTCATGAAGATCCTCACCGA |
|  | Reverse Primer | TTCTCCTTAATGTCACGCACGATT |
| MMP3 | Forward Primer | AGTCTTCCAATCCTACTGTTGCT |
|  | Reverse Primer | TCCCCGTCACCTCCAATCC |
| MMP9 | Forward Primer | TCTATGGTCCTCGCCCTGAA |
|  | Reverse Primer | CATCGTCCACCGGACTCAAA |
| ICAM-1 | Forward Primer | TGACCGTGAATGTGCTCTCC |
|  | Reverse Primer | TATGGGAAGGCCGAGGAAGA |
| NFE2L2 | Forward Primer | TCAGCGACGGAAAGAGTATGA |
|  | Reverse Primer | CCACTGGTTTCTGACTGGATGT |
| CXCL12 | Forward Primer | ATTCTCAACACTCCAAACTGTGC |
|  | Reverse Primer | ACTTTAGCTTCGGGTCAATGC |
| CXCR4 | Forward Primer | ACTACACCGAGGAAATGGGCT |
|  | Reverse Primer | CCCACAATGCCAGTTAAGAAGA |

1. Bonilla X, Vanegas NP, Vernot JP: **Acute Leukemia Induces Senescence and Impaired Osteogenic Differentiation in Mesenchymal Stem Cells Endowing Leukemic Cells with Functional Advantages.** *Stem Cells Int* 2019, **2019:**3864948.
